# GUN4 appeared early in cyanobacterial evolution

**DOI:** 10.1093/pnasnexus/pgad131

**Published:** 2023-04-12

**Authors:** Nathan C Rockwell, J Clark Lagarias

**Affiliations:** Department of Molecular and Cell Biology, University of California at Davis, One Shields Avenue, Davis, CA 95616, USA; Department of Molecular and Cell Biology, University of California at Davis, One Shields Avenue, Davis, CA 95616, USA

**Keywords:** Gloeobacterales, Thermostichales, ChlH

## Abstract

Photosynthesis relies on chlorophylls, which are synthesized via a common tetrapyrrole trunk pathway also leading to heme, vitamin B12, and other pigmented cofactors. The first committed step for chlorophyll biosynthesis is insertion of magnesium into protoporphyrin IX by magnesium chelatase. Magnesium chelatase is composed of H-, I-, and D-subunits, with the tetrapyrrole substrate binding to the H-subunit. This subunit is rapidly inactivated in the presence of substrate, light, and oxygen, so oxygenic photosynthetic organisms require mechanisms to protect magnesium chelatase from similar loss of function. An additional protein, GUN4, binds to the H-subunit and to tetrapyrroles. GUN4 has been proposed to serve this protective role via its ability to bind linear tetrapyrroles (bilins). In the current work, we probe the origins of bilin binding by GUN4 via comparative phylogenetic analysis and biochemical validation of a conserved bilin-binding motif. Based on our results, we propose that bilin-binding GUN4 proteins arose early in cyanobacterial evolution and that this early acquisition represents an ancient adaptation for maintaining chlorophyll biosynthesis in the presence of light and oxygen.

**Significance Statement**

Human societies rely on oxygenic photosynthesis, which uses sunlight and produces our oxygen, food, and fodder. Photosynthesis requires chlorophyll, and chlorophyll synthesis is performed by a series of enzymes including magnesium chelatase. However, light and oxygen can inactivate magnesium chelatase. It has been proposed that the GUN4 protein uses linear tetrapyrroles (bilins) to protect magnesium chelatase under these conditions. In this work, we study the evolution of GUN4 proteins and identify key amino acids that GUN4 uses to bind bilin. Our work shows that bilin-binding GUN4 proteins are widespread and ancient, implicating them as an essential early adaptation for oxygenic photosynthesis.

## Introduction

**Fig. 1.**
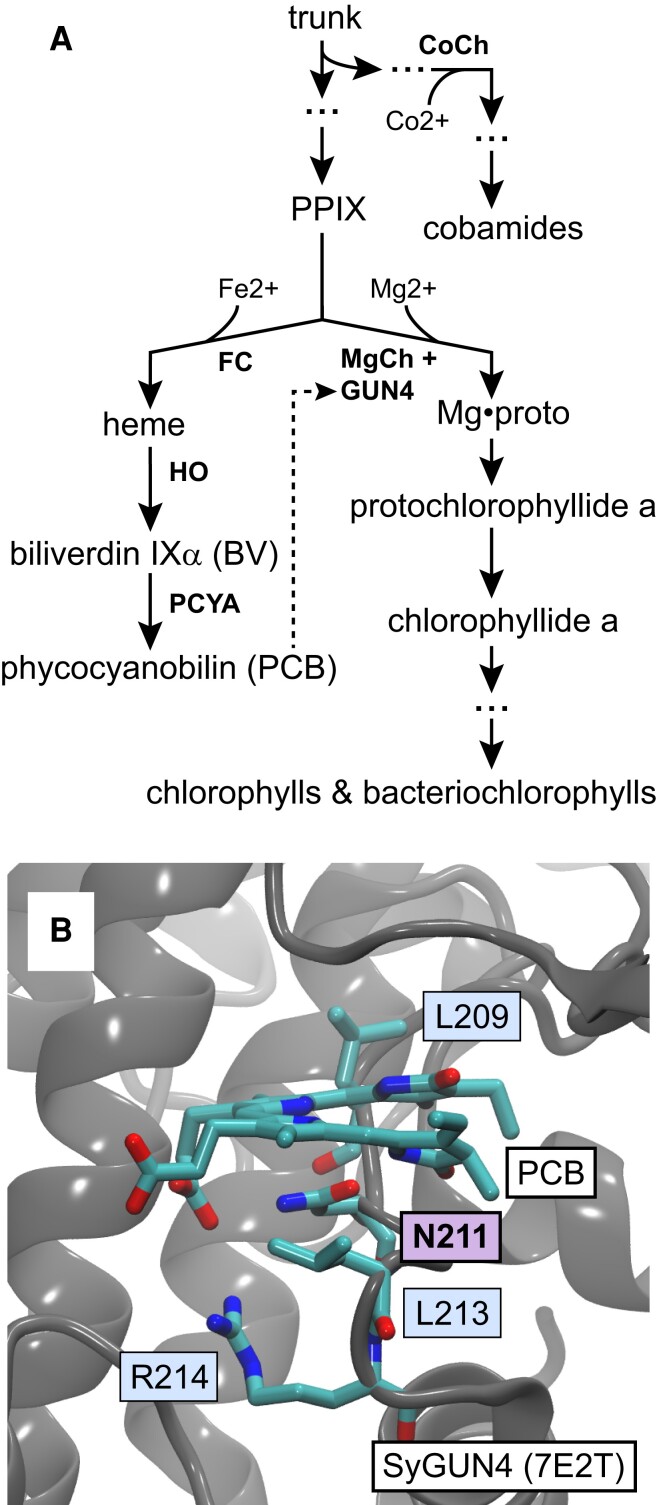
GUN4 and bilin in Chl biosynthesis. A) Heme, Chl, and cobamides such as vitamin B12 are synthesized via a common tetrapyrrole trunk pathway (1). Incorporation of iron into PPIX by FC produces heme and also provides a precursor for synthesis of bilins such as PCB. Incorporation of magnesium into PPIX by MgCh is the first committed step in Chl biosynthesis, producing Mg•protoporphyrin IX (Mg•proto). In oxygenic photosynthetic organisms, MgCh function is stimulated by GUN4 protein, which can bind PCB (dashed line). The equivalent step in cobamide biosynthesis is cobalt chelation by CoCh. In the aerobic pathway for cobamide synthesis, CoCh is composed of three subunits including a substrate-binding CobN subunit homologous to the substrate-binding subunit CHLH in MgCh. B) The structure of GUN4 from *Synechocystis* sp. strain PCC 6803 is shown with bound PCB (PDB accession 7E2T; (2)). The PCB ligand and a conserved LxNxLR motif are indicated.

Terrestrial ecosystems and human societies rely on oxygenic photosynthesis for carbon fixation and oxygen production. Both anoxygenic and oxygenic phototrophs utilize chlorophyll (Chl) or bacteriochlorophyll (Bchl), cyclic tetrapyrroles synthesized via a shared tetrapyrrole trunk pathway (1) that also gives rise to heme via protoporphyrin IX (PPIX; Fig. 1A). These two metalloporphyrin pathways diverge after PPIX, with chelation of different metals leading to either heme or Chls. Iron insertion by ferrochelatase (FC) gives rise to heme, which can in turn be metabolized into biliverdin IXα and then into phycobilins such as phycocyanobilin (PCB, Fig. 1A). FC can also generate zinc (Zn) porphyrins, which can be metabolized into Zn-Bchl to support anoxygenic photosynthesis (3–5).

As the first committed step of Chl synthesis, the chelation of magnesium into PPIX by magnesium chelatase (MgCh) is essential to all oxygenic photosynthetic species. MgCh is a large enzyme composed of H-, I-, and D-subunits, designated based on the host organism: CHLH, CHLI, and CHLD in eukaryotes; ChlH, ChlI, and ChlD in cyanobacteria; and BchH, BchI, and BchD in anoxygenic phototrophs. CHLH and CHLD are homologous to CobN and CobT of aerobic cobalt chelatase (CoCh), the enzyme responsible for cobalt insertion in the aerobic pathway for synthesis of vitamin B12 and related cobamide cofactors (6). In both enzymes, the large CHLH or CobN subunit is responsible for binding the tetrapyrrole substrate, while the other subunits power the reaction via ATP hydrolysis. Anaerobic BchH is rapidly inactivated under aerobic conditions by photoreaction of bound porphyrin in the presence of oxygen, resulting in covalently modified BchH protein (7). Hence, the oxygenation of the atmosphere during the great oxygenation event (GOE) approximately 2.4 billion years ago (Gya) required cyanobacteria to adapt MgCh to function in the presence of oxygen.

**Fig. 2.**
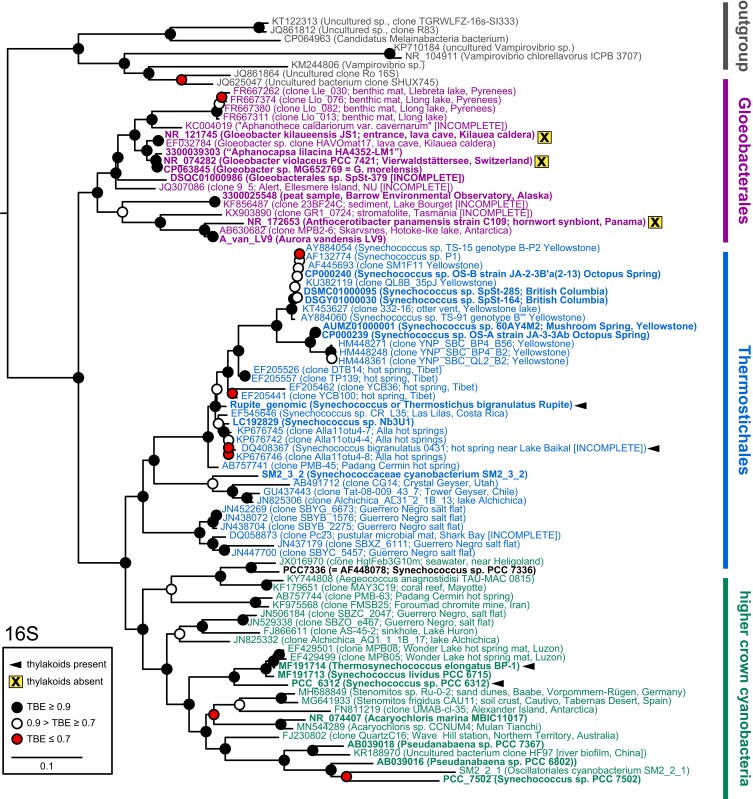
Phylogenetic analysis of 16S sequences implicates a G/T/HC topology for cyanobacterial evolution. A maximum likelihood phylogenetic tree is shown for 16S rDNA sequences from Gloeobacterales (mauve), Thermostichales (blue), higher crown cyanobacteria (green), and noncyanobacterial sequences (grey). This analysis demonstrates the G/T/HC topology, with Gloeobacterales as the earliest cyanobacterial branch and Thermostichales as the next branch (equivalent to the earliest branch containing thylakoids). Organisms known to contain thylakoids (arrowheads) or to lack thylakoids (boxed “X”) are indicated, and sequences with available genomes or MAGs are in bold face. The sequence from *Synechococcus* sp. strain PCC 7336 is indicated in black due to the ambiguous placement of this organism overall (see text). Root placement is between the outgroup (sequences from Melainabacteria) and all cyanobacterial sequences.

In oxygenic organisms, Mg chelation by MgCh is facilitated by an additional protein, GUN4 (Fig. 1A). First identified genetically as essential for proper regulation of nuclear genes in plants in response to chloroplast damage, GUN4 was later characterized biochemically as a small protein binding both the porphyrin substrate and product of MgCh; both plant and cyanobacterial MgCh activities were stimulated by GUN4 in vitro (8). Arabidopsis GUN4 is also able to bind to CHLH directly (9). In the cyanobacterium *Synechocystis* sp. strain PCC 6803 (hereafter, Synechocystis), the absence of GUN4 is associated with a range of phenotypes such as an inability to grow photoautotrophically under high light (10). More recent studies have established that GUN4 from Synechocystis and from the model green alga *Chlamydomonas reinhardtii* (hereafter, Chlamydomonas) can also bind linear tetrapyrroles (bilins) such as PCB (Fig. 1B; (2, 11)). PCB has no effects on MgCh activity alone, but it stimulates MgCh activity by more than an order of magnitude when bound to GUN4 (11). Loss of PCB biosynthesis in Chlamydomonas results in similar phenotypes to those of *gun4* mutants, confirming the importance of this property in vivo (11, 12). PCB is a core light harvesting component of cyanobacterial phycobilisomes, and its synthesis is essential in *Synechococcus* sp. PCC 7002 (13). Since phycobilisomes are not essential to cyanobacteria (14), PCB's role as a GUN4 cofactor appears critical for integrating multiple aspects of tetrapyrrole metabolism in oxygenic phototrophs.

GUN4 has been reported to be absent in *Gloeobacter violaceus* PCC 7421 (15), an early-diverging cyanobacterium lacking thylakoids (16–18). However, distant GUN4 homologs are detected in the genomes of *Gloeobacter* spp. by BLAST searches (19–21). Multiple GUN4 homologs are also found in Synechocystis (22). The closest Synechocystis relative to Arabidopsis GUN4 is the protein encoded by the *sll0558* gene, with that encoded by *sll1380* being the next closest relative (15). Phenotypic analysis demonstrated that loss of *sll0558* confers *gun4* phenotypes, whereas loss of *sll1380* does not (10, 15). GUN4 paralogs thus exist in cyanobacteria, underscoring the possibility that the distant GUN4 homologs in *Gloeobacter* spp. are also paralogs.

The existence of GUN4 paralogs is relevant to questions about the origins and functions of GUN4 itself: did it evolve from such paralogs, or the other way round? Is bilin binding widespread in GUN4 orthologs? Is it absent in paralogs? Early evolution of GUN4 orthologs in ancestral cyanobacteria and stimulation of MgCh activity by bilin could have been ancient adaptations to permit MgCh function in the presence of light and rising oxygen levels. The subsequent loss of GUN4 in *Gloeobacter* spp. might then reflect lack of selective pressure for GUN4 function in a slow-growing organism adapted for growth in low light. However, analyzing the origins of GUN4 poses challenges for phylogenetic analysis. GUN4 is apparently confined to oxygenic photosynthetic organisms, meaning that there is no clearly ancestral outgroup. Paralogs would provide such an outgroup if they are ancestral, but this is effectively circular reasoning.

We examine these questions in the current work by placing GUN4 in the context of early cyanobacterial evolution (Fig. S1). Recent studies demonstrate that the earliest known cyanobacterial branch, the Gloeobacterales, includes not only *Gloeobacter* spp. but also a range of other organisms (23–26). Within crown cyanobacteria, a range of isolates from Yellowstone and other hot springs that have previously been assigned to the unicellular genus *Synechococcus* (23, 27) have typically been recovered as the earliest branch, with this lineage sometimes also including the mesophilic isolate *Synechococcus* sp. PCC 7336 (Fig. S1; (28–31)). This Thermostichales lineage has recently been shown to contain thylakoids (27). Using phylogenetic analysis of 16S rDNA and of catenated ribosomal proteins, we extend this view by identifying early-branching mesophilic members from metagenomic studies of South African stromatolites (32). Several cyanobacterial genera have been repeatedly shown to be early branches within the subsequent cyanobacterial crown radiation, including *Pseudanabaena*, *Acaryochloris*, *Thermosynechococcus*, and *Gloeomargarita* (21, 23, 25–31, 33–35). Through comparison of this pattern of diversification to phylogenetic reconstructions of GUN4 and CHLH, we support the presence of both proteins in the last common cyanobacterial ancestor (LCCA) and imply that GUN4 paralogs arose later in cyanobacterial evolution. We also identify a single amino acid substitution within a conserved LxNxLR motif (Fig. 1B) that is sufficient to ablate PCB binding in Chlamydomonas GUN4 protein (CrGUN4). Phylogenetic analysis demonstrates that this motif is conserved in cyanobacterial GUN4 orthologs and in GUN4 proteins from many eukaryotic algae, but not in GUN4 paralogs. Taken together, these studies provide evidence that a bilin-binding GUN4 ortholog was present in LCCA, implying that the ternary complex of GUN4, bilin, and MgCh is an ancient adaptation for Chl biosynthesis in the presence of light and oxygen.

## Results

### A Gloeobacterales/Thermostichales/higher crown (G/T/HC) framework for early cyanobacterial evolution

GUN4 orthologs have been reported to be ubiquitous in oxygenic photosynthetic organisms except for the early-branching cyanobacterium *G. violaceus* PCC 7421 (15). This is consistent with the absence of such orthologs in subsequently isolated *Gloeobacter* spp. (21, 36), although distant homologs could be detected by BLAST (19) searches of both the *G. violaceus* and *Gloeobacter morelensis* genomes (20, 21). BLAST searches of other members of the Gloeobacterales (24, 26) with the functional Synechocystis GUN4 protein sll0558 (10, 15) identified possible orthologs. We therefore used GUN4 homologs from *Gloeobacter* spp., *Aurora vandensis* (24), and *Anthocerotibacter panamensis* (26) as queries against the assemblies from these other cyanobacterial species to obtain pairwise identities shown in Fig. S2. GUN4 homologs from *Gloeobacter* spp. were very close to each other and to a sequence from a metagenome-assembled genome (MAG) initially assigned to *Aphanocapsa lilacina* (37) but subsequently assigned to Gloeobacterales (25). These three sequences lacked close relatives in a broad range of other cyanobacteria, a pattern similar to that of the known Synechocystis GUN4 paralog sll1380 (Fig. S2; (15)). In contrast, the GUN4 homologs from *A. vandensis* and *A. panamensis* (Fig. S2) exhibited closer relatives in a broad range of cyanobacterial genomes and MAGs, including Synechocystis. These results suggest that GUN4 orthologs are present in *A. vandensis* and *A. panamensis*. The presence of GUN4 orthologs in these species could implicate an early origin of this protein in cyanobacteria, but it also raises the possibility that such sequences were later acquisitions arising via horizontal gene transfer (HGT). To address this question, we sought to compare the phylogenetic analysis of GUN4 with the pattern of early cyanobacterial evolution.

Previous phylogenomic studies have provided support for Gloeobacterales as the earliest branch in the process of cyanobacterial diversification (Fig. S1), consistent with the lack of thylakoids in this lineage and the presence of thylakoids in all other cyanobacterial lineages (18, 26, 27, 36, 38). Such phylogenomic studies provided good support for Thermostichales as the earliest branch within crown cyanobacteria and also identified several other early-branching crown cyanobacterial lineages without consensus on the precise order of branching for those lineages (Fig. S1). Hence, these studies establish a well-supported G/T/HC (Gloeobacterales/Thermostichales/higher crown) topology for cyanobacterial evolution despite the lack of a robust view of subsequent branching within the higher crown. We began by analyzing 16S rDNA sequences, including a fragmentary 16S sequence from Gloeobacterales sp. SpSt-379 (hereafter, SpSt-379), a MAG recently assigned to Gloeobacterales (21, 25). The resulting tree recovered a well-defined Gloeobacterales clade that includes this sequence (Fig. 2, with additional information in Table S1). Consistent with previous work (25, 26), we observed two clades within Gloeobacterales: a clade including *Gloeobacter* spp. sequences and another including *A. vandensis* and *A. panamensis* sequences. The first clade includes known *Gloeobacter* 16S sequences as well as one originally assigned to *Aphanocapsa caldariorum* var. *cavernarum*, but later shown to be closely related to *Gloeobacter* (23). This clade also includes five sequences associated with genomes or MAGs: SpSt-379, *G. violaceus*, *Gloeobacter. kilaueensis*, *G. morelensis*, and the MAG originally assigned to *A. lilacina* (hereafter, “*A. lilacina*;” (25, 37)). Previous work with multiprotein trees (25) placed two additional MAGs in this clade (ES-bin-141 and ES-bin-313), but 16S sequences could not be found in these assemblies. The second clade includes sequences associated with three genomes or MAGs: *A. vandensis* LV9, *A. panamensis*, and a MAG from Alaskan peat previously assigned to this branch of Gloeobacterales (Fig. 2; (25)). An additional MAG assigned to this clade (24, 25), *A. vandensis* MP9P1, possesses a fragmentary 16S sequence perfectly matching the longer sequence of *A. vandensis* LV9; this sequence was omitted. Other 16S sequences in the Gloeobacterales lineage are from a range of environments, including the Arctic, the Antarctic, European mountains, and Tasmanian stromatolites (Fig. 2). As expected, all three cyanobacterial species known to lack thylakoids were recovered within the Gloeobacterales.

The earliest branch within crown cyanobacteria, the Thermostichales (27), included 16S sequences from thermophilic *Synechococcus* isolates from Yellowstone and from Rupite hot spring, Bulgaria (Fig. 2). This clade also included additional sequences from hot springs in British Columbia, Japan, Tibet, Russia, and Indonesia. Genomes or MAGs are available for isolates from Yellowstone and British Columbia (39, 40). Additionally, genomes are available for *Synechococcus bigranulatus* Rupite (also *Thermostichus bigranulatus* or *Thermostichus vulcanus* (27, 41)) and for *Synechococcus* sp. strain Nb3U1 from Japan (42). The presence of thylakoids has been demonstrated for both the Rupite strain (27) and *S. bigranulatus* 0431 from Russia (43), which does not have a genome but whose 16S sequence was placed with other hot springs isolates. We also identified two early-diverging lineages of cyanobacteria within this clade (Fig. 2). No genomes or MAGs could be found for the earliest lineage, but the second group included a 16S sequence associated with a MAG (hereafter, SM2_3_2) from a South African stromatolite (32). Overall, the composition of this branch is consistent with the proposed Thermostichales lineage (27) with the incorporation of early-branching mesophilic members but with the exclusion of *Synechococcus lividus* PCC 6715. In our analysis and in another recent study (31), *S. lividus* PCC 6715 was instead associated with *Thermosynechococcus* spp. in the third cyanobacterial clade, the higher crown cyanobacteria (Fig. 2). This clade included several branches with available genomes and MAGs, including members of *Thermosynechococcus* and *Acaryochloris* as well as the filamentous genus *Pseudanabaena*. The higher crown cyanobacterial clade also included *Synechococcus* sp. PCC 7336, which was clustered with the Yellowstone isolates in earlier analyses (28–31, 33). Our 16S analysis thus recovers the expected G/T/HC topology for early cyanobacterial evolution.

**Fig. 3.**
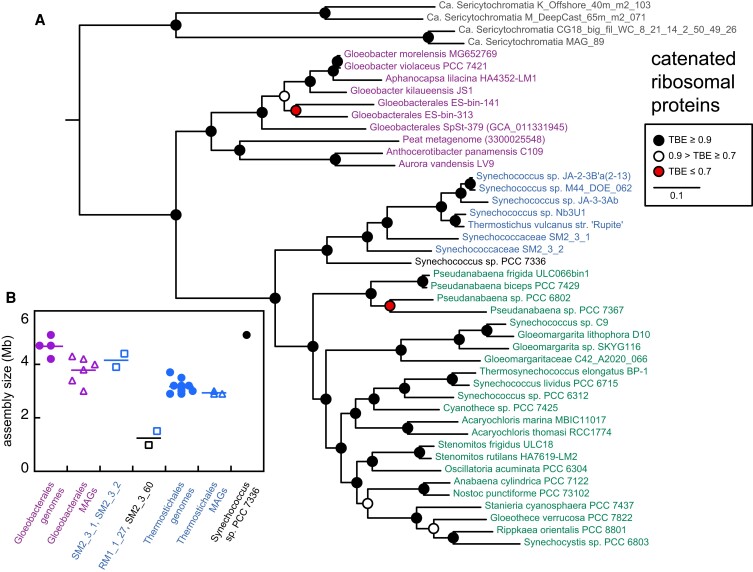
Identification of early-branching members of Thermostichales using catenated ribosomal proteins. A) A maximum likelihood phylogenetic tree is shown for catenated ribosomal proteins in the scheme of Fig. 2. Assemblies used for constructing the catenated sequence alignment are indicated in Fig. S3 except for Gloeobacterales SpSt-379 and a Gloeobacterales MAG from Arctic peat, both of which are listed here and were assigned to Gloeobacterales previously (25). The catenation comprised eight proteins: S5, L15, L13, S9, S4, L1, S7, and L9, in that order. Details of constructing the catenated sequence alignment are presented in the Supplemental Text. Root placement is between the outgroup (four Sericytochromatia MAGs) and all cyanobacterial sequences. B) Assembly size is plotted for genomes and MAGs from Gloeobacterales, Thermostichales, and other organisms. Sequenced genomes are shown in filled circles, MAGs from South African stromatolites are shown as open squares, and other MAGs are shown as open triangles.

The identification of a potential early, mesophilic branch within Thermostichales represented by SM2_3_2 prompted us to seek other potential members of this lineage. BLAST searches with a range of protein sequences from SM2_3_2 identified three other MAGs from the same study as potential close relatives: SM2_3_1, RM1_1_27, and SM2_3_60. As 16S sequences could not be detected in these MAGs, we used a set of 23 proteins of ≥128 amino acids from *Thermosynechococcus elongatus* (Table S2) to design ribosomal protein catenations for phylogenetic analysis. Ribosomal proteins were poorly represented in RM1_1_27, with only 4 out of the 23 detected (Table S2). Three of these proteins were identical to protein sequences from SM2_3_2, so RM1_1_27 sequences were not used in further analysis. Nine complete sequences were found in SM2_3_60, but these poorly overlapped with those of SpSt-379 (Table S2). We therefore constructed two catenations of ribosomal proteins for phylogenetic analysis, using sequences from the nonphotosynthetic cyanobacterial relative Sericytochromatia (44) as an outgroup. Phylogenetic analysis of both catenations (Figs. 3A and S3) recovered the G/T/HC topology, with SM2_3_1 and SM2_3_2 placed as early branches within Thermostichales. *Synechococcus* sp. PCC 7336 was placed as either the earliest branch within Thermostichales or as a sister lineage. SM2_3_60 was placed in a clade with *Synechococcus* sp. PCC 7336 (Fig. S3). The MAGs SM2_3_1 and SM2_3_2 are at least as large as genomes from their thermophilic relatives (Fig. 3B), suggesting that their complete genomes may be similar in size to that of *Synechococcus* sp. PCC 7336. RM1_1_27 and SM2_3_60 are much smaller MAGs, consistent with the poorer representation of ribosomal proteins in these assemblies (Fig. 3B and Table S2). Taken together, these ribosomal protein phylogenies corroborate the G/T/HC topology from 16S sequences, place SM2_3_1 and SM2_3_2 within that topology, and provide a good framework for comparison to phylogenetic analysis of GUN4.

**Fig. 4.**
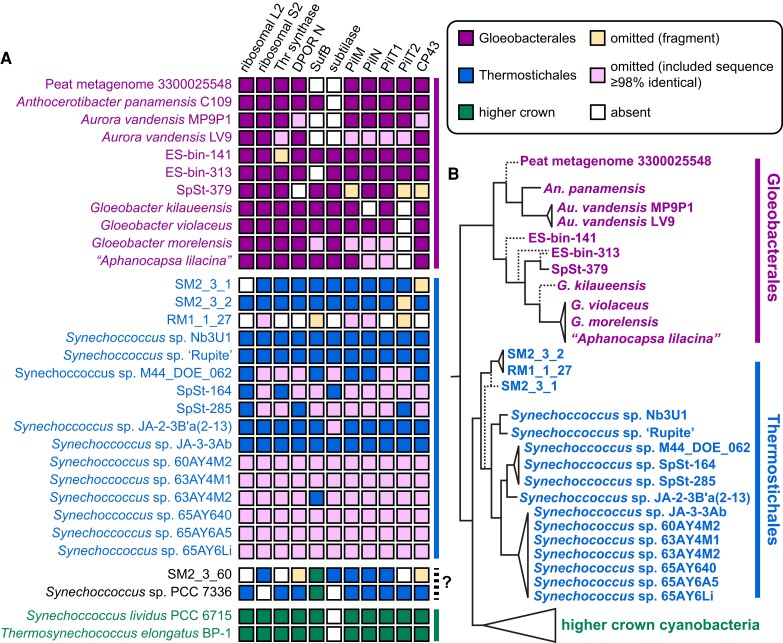
Validation of the G/T/HC topology using individual genes. A) The presence or absence of sequences in individual genomes and MAGs is indicated, along with their assignment to Gloeobacterales, Thermostichales, or higher crown clades using the scheme of Fig. 2. Each sequence was analyzed individually by phylogenetic analysis (Figs. S4–S13). *Synechococcus* sp. PCC 7336 and MAG SM2_3_60 from a South African stromatolite (32) were ambiguously placed within either Thermostichales or higher crown cyanobacteria. *S. lividus* PCC 6715 was robustly placed within higher crown cyanobacteria. B) The 10 individual single-protein trees were compared (Fig. S14) to construct an approximate topology for early diversification of cyanobacteria, allowing comparison to trees calculated using 16S sequence (Fig. 2) or catenated ribosomal proteins (Figs. 3 and S2). Branches supported by ≥70% of the individual trees are indicated as solid lines; other branches are indicated as dashed lines. A maximum of eleven proteins could be evaluated for each node, because of the presence of PilT1 and PilT2 isoforms (Fig. S12), but missing or partial sequences reduced the actual number in most cases. ES-bin-313 and SpSt-379 had particularly poor overlap within this set (*n* = 6: Fig. S14), so two possible topologies are indicated. SM2_3_1 and SM2_3_2 were reliably placed as one or more early branches within Thermostichales, but whether they comprise a single branch was less clear. Both possibilities are indicated.

We next inferred maximum likelihood phylogenies for a series of single-protein trees varying in size and sampling as closer parallels to analysis of GUN4. Rather than attempting to span known diversity in the higher crown clade, we largely focused on early branches in the higher crown cyanobacteria, such as *Pseudanabaena* or *Acaryochloris*. We began with ribosomal proteins L2 (Fig. S4) and S2 (Fig. S5), which were not included in either catenation. We next tested threonine synthase (Fig. S6), focusing on a particular isoform (protein accession HGZ88566) that was also present on the scaffold containing the 16S sequence of Gloeobacterales sp. SpSt-379. We subsequently examined a series of proteins varying in size and in availability in Gloeobacterales and Thermostichales (Fig. 4A and Table S1): the N subunit of dark-operative protochlorophyllide reductase (DPOR N subunit; Fig. S7); the ABC transporter subunit SufB (Fig. S8); Ca^2+^–dependent serine proteases of the subtilisin or peptidase S8 family (subtilases; Fig. S9); the PilM (Fig. S10), PilN (Fig. S11), and PilT (Fig. S12) proteins implicated in the function of type IV pili; and the Chl-binding protein CP43 (Fig. S13). These proteins provide different parallels to GUN4. The DPOR N subunit is part of a multiprotein complex carrying out one step in Chl biosynthesis. SufB is also part of a multiprotein complex, but it is not well represented in Gloeobacterales (Fig. 4A). Subtilases were absent in the branch of the Gloeobacterales including *A. vandensis* and *A. panamensis* (Fig. 4A), so SufB and subtilases are similar to poor representation of GUN4 orthologs in Gloeobacterales. Proprotein convertases (45) provided an outgroup for subtilases, so we also included sequences from more derived cyanobacteria in this analysis. PilN is a small protein, comparable in size to GUN4 (Table S1). There is a functional distinction between PilT1 and PilT2 isoforms in *Nostoc punctiforme* (46) that is reminiscent of the split between GUN4 orthologs and paralogs, so sequences from derived cyanobacteria were also included in the PilM, PilN, and PilT analyses. Like GUN4, CP43 is confined to oxygenic photosynthetic organisms, meaning there is no reliable outgroup. In all cases, the G/T/HC topology was recovered, with SM2_3_1 and SM2_3_2 sequences placed within the Thermostichales clade. SM2_3_60 and *Synechococcus* sp. PCC 7336 sequences were often placed within the Thermostichales clade or sister to it, but these sequences were instead placed within higher crown cyanobacteria in the analysis of SufB (Figs. 4A and S8). A meta-analysis of these single-protein trees (Fig. S14) recovered a topology for Gloeobacterales and Thermostichales (Fig. 4B) that largely matches recent work (25, 26). Within Thermostichales, we clearly recover the mesophilic cases from South Africa as one or more early branches, followed by genomes and MAGs from hot spring environments. Taken together, these studies demonstrate three key points: one, that the expected G/T/HC topology (Fig. S1) can be recovered in phylogenetic analysis of single proteins of varying size; two, that this topology can be recovered in the presence of multiple isoforms and without sequences from some early-branching organisms; and, three, that this topology can be recovered without an outgroup. It thus seemed appropriate to examine GUN4 using the same approach.

### Phylogenetic analysis of GUN4 and CHLH reveals both to be ancestral cyanobacterial genes

**Fig. 5.**
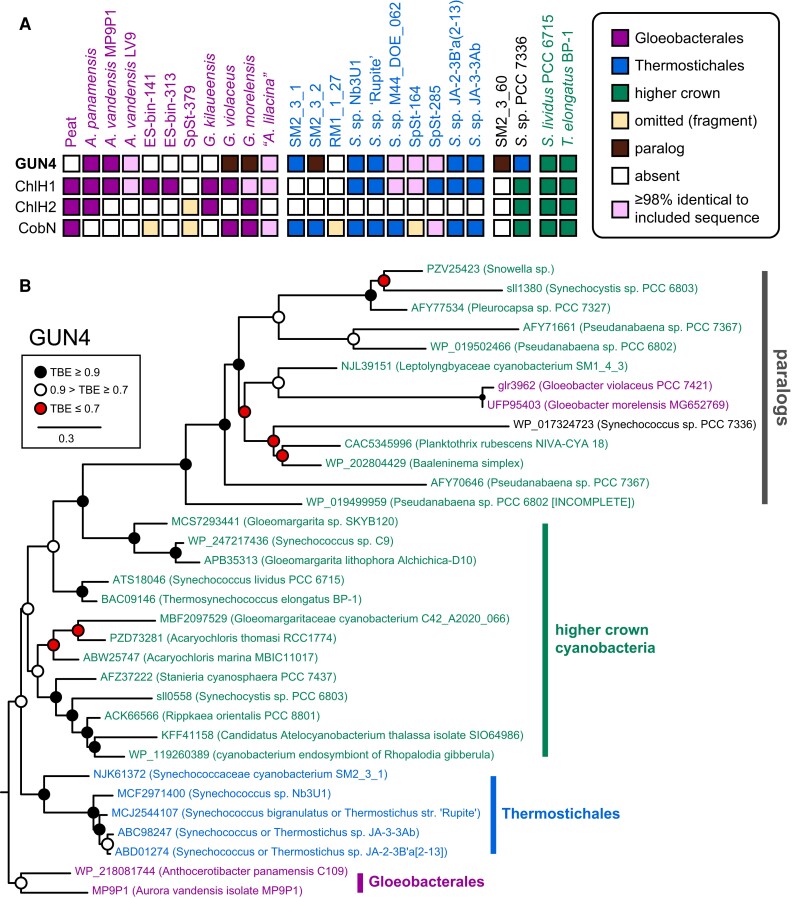
Phylogenetic analysis of GUN4 is consistent with the G/T/HC topology. A) The availability of GUN4, CHLH, and CobN sequences in available genomes and MAGs of Gloeobacterales and Thermostichales is shown. B) A maximum likelihood phylogenetic tree is shown for GUN4, following the conventions of Fig. 2. An apparent clade of paralogs was defined based on the presence of sll1380 from Synechocystis (15). Root placement is between orthologs from Gloeobacterales and all other sequences.

Within Gloeobacterales, GUN4 homologs are only present in *A. panamensis*, *A. vandensis*, *G. violaceus*, *G. morelensis*, and “*A. lilacina”* (Figs. 5A and S2). The lack of GUN4 sequences in other Gloeobacterales species (Fig. 5A) may reflect incomplete coverage, consistent with the absence of other sequences from such MAGs (Fig. 4A). However, the complete absence of GUN4 homologs in the *G. kilaueensis* genome is notable. Within Thermostichales, by comparison, GUN4 homologs are ubiquitous in hot springs genomes and MAGs (Fig. 5A). Homologs are also found in SM2_3_1, SM2_3_2, and SM2_3_60 but are missing in the less complete RM1_1_27. Multiple homologs are present in *Synechococcus* sp. 7336. Examination of the pairwise identities of these sequences with a range of cyanobacterial assemblies (Fig. S2) suggests that GUN4 homologs from SM2_3_2 and SM2_3_60 are paralogs, whereas the sequence from SM2_3_1 is likely to be an ortholog.

We therefore constructed a GUN4 sequence alignment including sequences from Gloeobacterales, Thermostichales, and a range of higher crown cyanobacteria, including the known GUN4 ortholog and paralog from Synechocystis (15). We initially omitted one sequence from *Synechococcus* sp. PCC 7336 due to the variable placement of this organism in different phylogenetic analyses (Figs. 2, 3, and S3–S13). We also initially omitted candidate GUN4 orthologs from *Pseudanabaena* spp., because inclusion of these sequences with the initial group of paralogs resulted in a lower quality alignment with more gap-enriched positions. The resulting alignment was used to infer a maximum likelihood phylogeny. Using this tree, we evaluated two scenarios. In the first scenario (Fig. 5B), we assumed that a GUN4 ortholog was present in LCCA and that GUN4 paralogs evolved later via duplication and subsequent loss of GUN4 function. This implies placement of a root between Gloeobacterales orthologs and all other sequences. Such root placement does recover the expected G/T/HC topology for GUN4 orthologs (Fig. 5B), with the appearance of paralogs as a loosely supported, derived clade including the known paralog sll1380 from Synechocystis along with sequences from *G. violaceus* and *G. morelensi*s. The known Synechocystis GUN4 ortholog sll0558 is part of a paraphyletic grade of GUN4 sequences from higher crown cyanobacteria. The GUN4 homolog from SM2_3_1 is placed in the ortholog clade, as expected (Fig. S2), and is an early branch within a clade of Thermostichales GUN4 orthologs that matches the overall pattern of evolution within these organisms (Figs. 3 and 4B). Interestingly, GUN4 orthologs from *Gloeomargarita lithophora* and related organisms such as *Synechococcus* sp. C9 appear most closely related to the paralogs. Overall, the hypothesis that GUN4 is an ancestral cyanobacterial gene is compatible with the observed tree (Fig. 5B).

**Fig. 6.**
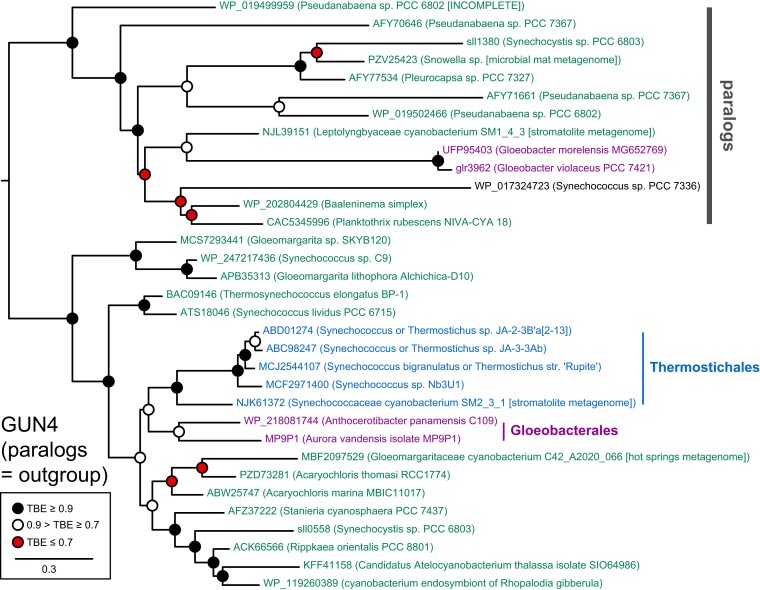
Alternate scenario for GUN4 evolution. The phylogenetic tree from Fig. 5B is shown with an alternate root placement between GUN4 orthologs and GUN4 paralogs.

In the alternate scenario (Fig. 6), we assumed that GUN4 orthologs evolved from paralogs via duplication and gain of function, implying a root placement between these two groups. In this hypothesis, GUN4 orthologs would have arisen within higher crown cyanobacteria. However, the G/T/HC topology was not well supported in the paralogs. The appearance of GUN4 orthologs in some members of the Gloeobacterales and in the Thermostichales would indicate that these lineages acquired GUN4 after their divergence from other cyanobacteria, implying more recent introduction of GUN4 orthologs via HGT in these lineages. This topology does retain a well-supported clade of GUN4 sequences from Thermostichales, implying an HGT event into the common ancestor of this taxon after they diverged from other cyanobacteria but while that ancestor remained a single genetic population. Such a scenario effectively implies an apparent discontinuity in which Thermostichales branched from other crown cyanobacteria before emergence of GUN4 but remained a single genetic population for some time thereafter.

We next asked whether this analysis could be recovered using other techniques and with inclusion of the omitted sequences. We first used the same protein sequence alignment to calculate a phylogeny using Bayesian inference from randomly generated starting trees. The resulting tree is shown compared with the maximum likelihood tree as a tanglegram in Fig. S15, using the root placement of Fig. 5B. The two trees are very similar, with the G/T/HC topology recovered for GUN4 orthologs in both cases and with identical Thermostichales clades that each include the GUN4 ortholog from SM2_3_1 as the earliest branch. The relationships in the paralog clade show more significant differences in the two analyses, but its composition and placement as sister to GUN4 from *Gloeomargarita* and related organisms were identical in both trees. We next carried out a maximum likelihood analysis of the DNA coding sequences for these proteins. The resulting phylogeny is again shown as a tanglegram with the maximum likelihood analysis of the amino acid sequences (Fig. S16), using the same root placement. Excepting slight differences in the branching order of the hot springs isolates of Thermostichales, the DNA-based tree adopts a branch pattern also seen in ribosomal protein S2 (Fig. S5) rather than the consensus branching order (Figs. 2, S3, and S14). There are also more substantial changes in the paralog clade (Fig. S16). Lastly, we included candidate orthologs from either *Synechococcus* sp. PCC 7336 or *Pseudanabaena* spp., each requiring slightly different sampling of GUN4 paralogs to retain a comparable number of characters in the final sequence alignment (Table S1). The resulting trees (Figs. S17–S18) confirm these sequences as orthologs and place them within higher crown cyanobacteria. All of these trees support four key conclusions: one, there is a clear separation of GUN4 orthologs and paralogs; two, the orthologs follow the G/T/HC topology of early-branching cyanobacteria; three, the Thermostichales GUN4 orthologs behave as though they were vertically inherited from the common ancestor of this lineage; and, four, orthologs from *Gloeomargarita* species, *Synechococcus* sp. C9, and related organisms are the protein sequences most closely related to the paralogs.

**Fig. 7.**
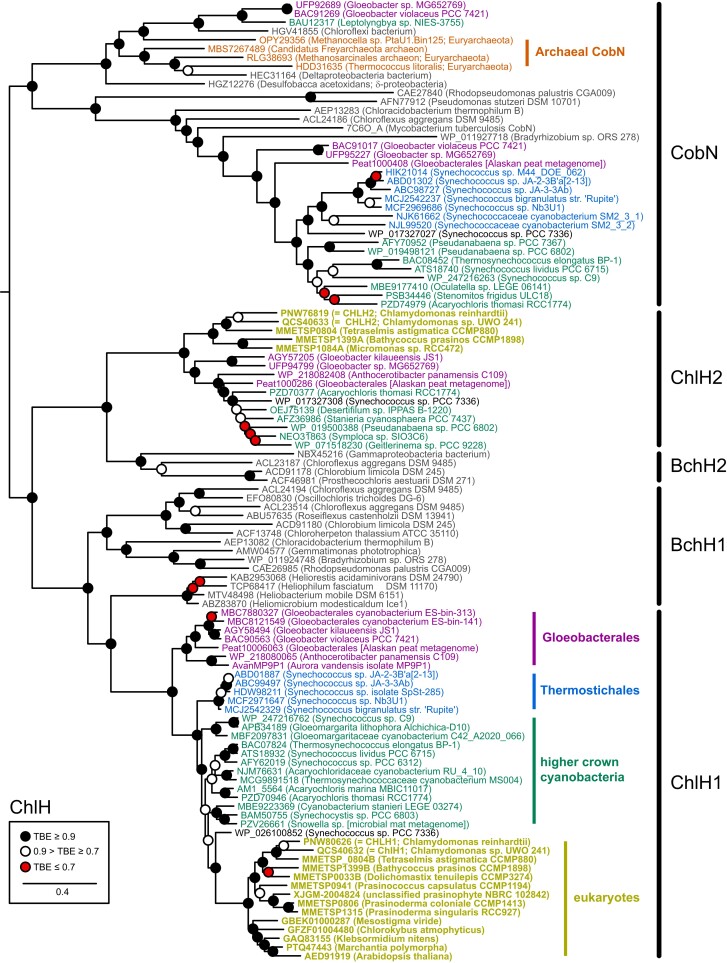
Phylogenetic analysis of ChlH and CobN. A maximum likelihood phylogenetic tree is shown for CHLH and BchH, using the conventions of Fig. 2 with additional eukaryotic CHLH sequences and archaeal CobN sequences. BchH1/CHLH1 and BchH2/CHLH2 isoforms are indicated, as is the G/T/HC topology for CHLH1. The root is placed between CobN and ChlH proteins.

If GUN4 orthologs are derived from GUN4 paralogs, then vertical inheritance of GUN4 orthologs in Thermostichales would imply a discontinuity between the emergence of this lineage and its acquisition of GUN4 (see above). Given that the function of GUN4 orthologs relies on their interaction with CHLH (9), we performed phylogenetic analysis of ChlH/BchH proteins to see if there was any evidence for a similar discontinuity in evolution of this GUN4-interacting protein. Unlike the analysis of GUN4, phylogenetic analysis of ChlH benefits from the presence of BchH orthologs in anoxygenic photosynthetic bacteria and from the availability of the CobN subunit of CoCh as a clear outgroup. However, there are two ChlH/CHLH isoforms present in some cyanobacteria and green algae, with the ChlH2 isoform apparently absent in Thermostichales (Fig. 5A). To aid in assigning the ChlH/CHLH isoforms, our phylogenetic analysis thus included green algal CHLH1 and CHLH2 sequences together with their bacterial relatives and prokaryotic CobN sequences. The inferred ChlH/CobN tree (Fig. 7) shows that the two ChlH isoforms arose as BchH1 and BchH2 isoforms in anoxygenic photosynthetic bacteria. CobN itself is only poorly represented in Gloeobacterales (Fig. 5A) and was found in two distinct clades. One group of cyanobacterial CobN sequences was found in some *Gloeobacter* spp. and only a few other cyanobacteria. These sequences were related to archaeal sequences and were only distantly related to other cyanobacterial CobN sequences. The more common group of Gloeobacterales CobN proteins is part of a clade also including CobN from Thermostichales and from higher crown cyanobacteria. This clade is derived from CobN sequences from other bacteria. Therefore, we conclude that cyanobacterial ChlH and CobN proteins arose independently from BchH and CobN ancestors, followed by an early duplication of BchH proteins. ChlH2/CHLH2 was recovered in separate prasinophyte/chlorophyte and cyanobacterial clades, but it is less common than BchH1/ChlH1 and is absent in several important cyanobacteria, including the Thermostichales (Fig. 5A) and *G. lithophora*. CHLH1 is well represented in Gloeobacterales and in thermophilic members of the Thermostichales, and each of these groups was recovered as a separate clade. CHLH1 from higher crown cyanobacteria was recovered as a paraphyletic grade, with eukaryotic CHLH1 descended from the higher crown sequences. The presence of Thermostichales CHLH1 sequences thus allows us to examine the evolution of the H1 isoform with higher confidence, and the G/T/HC topology was clearly recovered in ChlH1 without any apparent discontinuities. Hence, we found no evidence for subsequent acquisition of extant Thermostichales CHLH sequences via HGT, and CHLH1 clearly follows the G/T/HC topology. Taken together (see “Discussion”), these data favor the scenario with the simplest interpretation: GUN4 is an ancestral cyanobacterial gene.

### Conservation of a bilin-binding motif in GUN4 evolution implicates a bilin-dependent mode of action

**Fig. 8.**
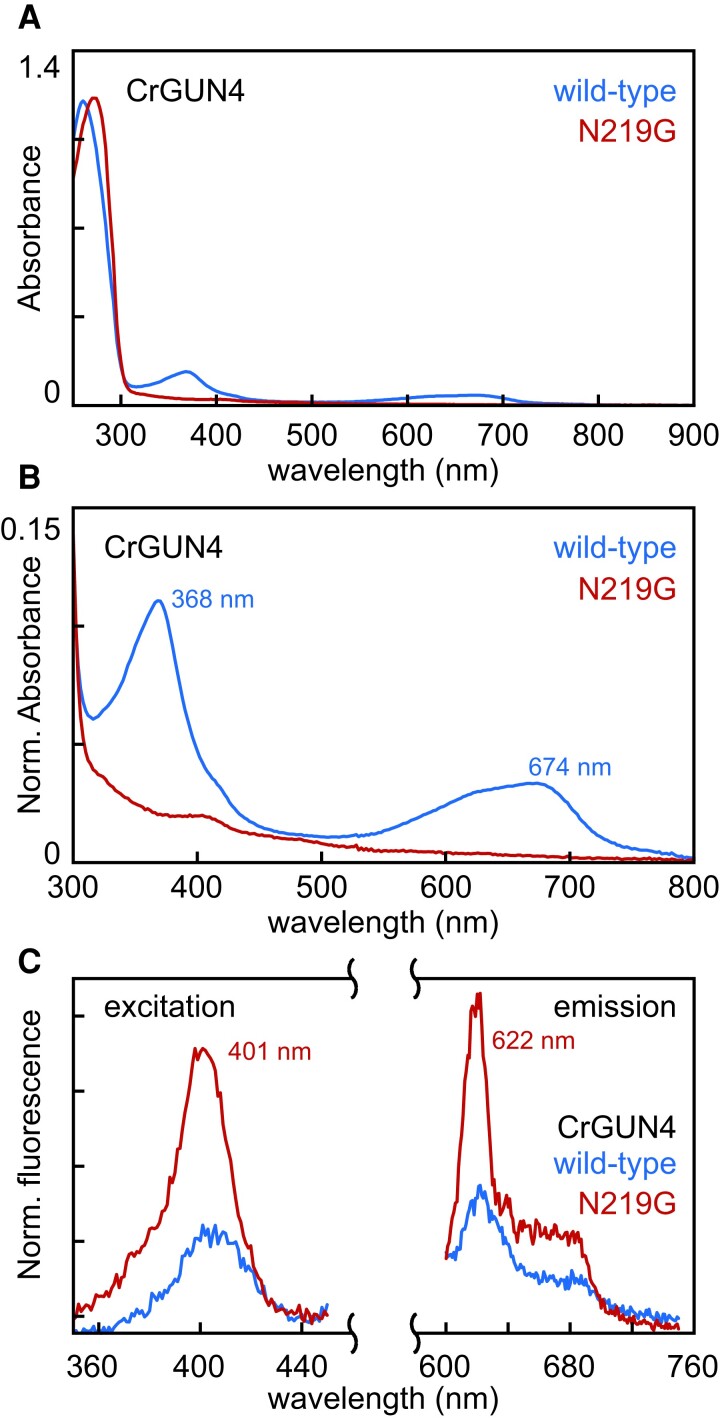
Spectroscopic characterization of recombinant GUN4 proteins. A) Absorption spectra are shown for wild-type and N219G CrGUN4 protein after heterologous expression in *E. coli* cells synthesizing PCB and purification (11, 48). B) A detail view of the spectra from panel A is shown, with peak wavelengths indicated for the wild type. C) Fluorescence excitation (left) and emission (right) spectra are shown, using the scheme of panel A. Excitation spectra were recorded at an emission wavelength of 620 nm, and emission spectra were recorded at an excitation wavelength of 404 nm. Peak wavelengths are indicated for the N219G variant, and fluorescence was normalized to the peak absorption on the aromatic amino acid band for each preparation (shown in panel B).

In parallel with phylogenetic analyses, we examined conservation of the bilin-binding pocket in GUN4 evolution. PCB is known to bind to GUN4 from both Synechocystis and Chlamydomonas. Conservation of bilin binding can thus provide independent confirmation of well-defined clades of GUN4 orthologs and paralogs as well as insight into the potential conservation of bilin binding in the largely uncharacterized GUN4 proteins from diverse eukaryotic algae. We began by examining the known crystal structure of GUN4 from Synechocystis with bound PCB (Fig. 1B). This analysis revealed the presence of an LxNxLR motif proximal to the bilin, with Asn211 on the β face of the PCB π system (47) in close proximity to the B- and C-rings. Asn211 corresponds to Asn219 in Chlamydomonas GUN4. By contrast with the Synechocystis GUN4 structure with bound bilin, part of this motif (Leu221 and Arg222) is not resolved in the apoprotein structure of Chlamydomonas GUN4 (2), consistent with ordering of this motif upon ligand binding. Unlike Synechocystis GUN4 (2), CrGUN4 exhibits PCB incorporation upon recombinant expression in *Escherichia coli* cells engineered to synthesize PCB (11, 48). This property allowed us to examine tetrapyrrole binding by wild-type and N219G CrGUN4 with this expression system. The N219G variant was chosen based on the presence of Gly at this position in the apparent *G. violaceus* GUN4 paralog glr3962. Wild-type CrGUN4 contains a small amount of bilin as shown by characteristic peaks in the absorbance spectrum at 368 and 674 nm, but these peaks are absent in the N219G variant (Fig. 8A and B). Interestingly, the loss of bilin binding in this variant does not prevent porphyrin binding. As shown by fluorescence spectroscopy, the relative intensity of porphyrin fluorescence per protein absorbance on the aromatic amino acid band is higher in the N219G variant than in WT (Fig. 8C). Therefore, this residue modulates the specificity of CrGUN4 for tetrapyrroles, confirming the importance of this motif. However, even this non-conservative substitution does not ablate porphyrin binding and hence does not preclude folding.

**Fig. 9.**
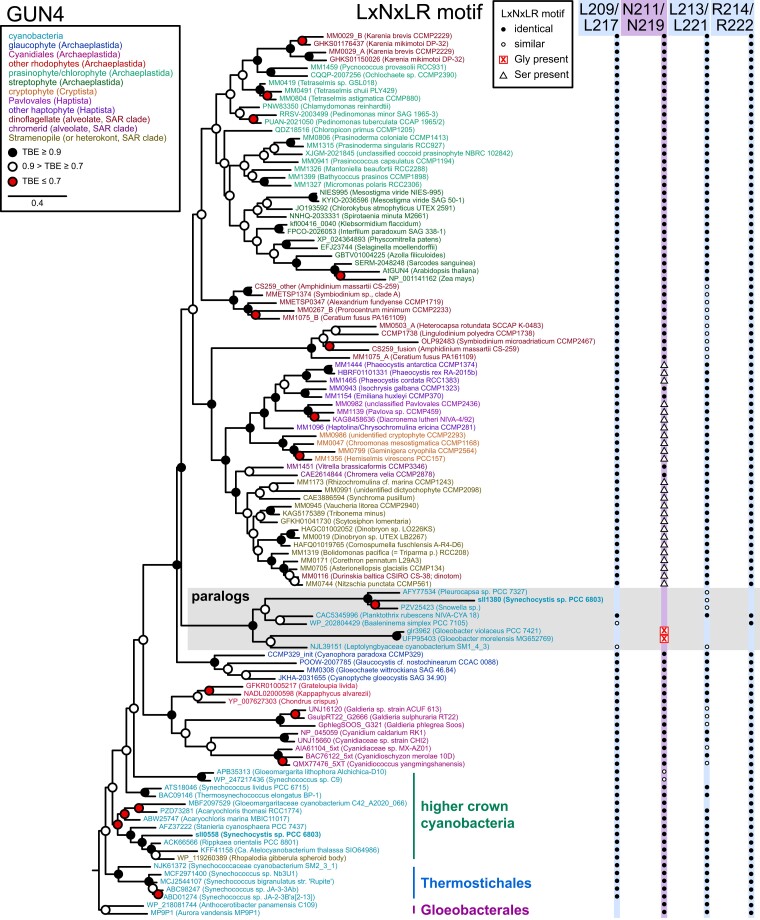
Conservation of the LxNxLR motif in eukaryotic GUN4 proteins. A maximum likelihood phylogenetic tree is shown for GUN4. Root placement matches that of Fig. 5B, but eukaryotic GUN4 sequences are included and proteins are coded by taxon as indicated in the legend. The G/T/HC topology is indicated, as are paralogs (box). On the right, conservation of each residue in the LxNxLR motif is indicated: filled circle, identity; open circle, conservative substitution. The presence of Gly (boxed “X”) or Ser (open triangle) in place of Asn219 is indicated. Conservative substitutions were defined as Ile, Val, or Met for Leu and Asp for Asn.

To examine conservation of this motif in eukaryotic algae, we next inferred a maximum likelihood GUN4 phylogeny including both cyanobacterial and eukaryotic sequences (Fig. 9). Overall statistical confidence was much lower in this analysis, so we also carried out a Bayesian analysis of the same proteins (Fig. S19). The two analyses are in good overall agreement. Several points indicate that this approach provides a useful framework for examining bilin binding. First, the expected G/T/HC topology for presumptive cyanobacterial GUN4 orthologs was recovered. Second, cyanobacterial GUN4 paralogs comprise a later branch, as expected. Both analyses place GUN4 paralogs as branching after appearance of rhodophyte GUN4, implying that these paralogs appeared at or after the time of primary endosymbiosis. Glaucophyte GUN4 sequences are also consistent with this interpretation. The late emergence of the paralog clade is also consistent with its placement as sister to *Gloeomargarita* species and related organisms in the cyanobacterial GUN4 tree (Fig. 5B), because *G. lithophora* is the closest known relative to the modern plastid (29). All other eukaryotic sequences emerge as a single clade after the paralog branch in both trees (Figs. 9 and S19). GUN4 from Viridiplantae (green algae and land plants) is recovered as a clade also including GUN4 from the dinoflagellate genus *Karenia*, which contains a tertiary, haptophyte-derived plastid (49). GUN4 sequences from most algae with rhodophyte-derived plastids comprise a clade sister to the green algal clade that is distinct from rhodophyte GUN4. This clade includes haptophyte, cryptophyte, chromerid, and stramenopile sequences, along with a group of dinoflagellate sequences which were found as CHLH fusion transcripts. Other non-chimeric dinoflagellate sequences comprise a small clade that was variably placed in the two analyses. GUN4 from the dinotom *Durinskia baltica* is not associated with other dinoflagellate sequences but is instead found within diatom GUN4 sequences, indicating that it is supplied by the diatom-derived plastid in this organism (49, 50). The early divergence of rhodophyte GUN4 and subsequent divergence of glaucophyte GUN4 is consistent with some recent studies on divergence of these organisms (51, 52), although the lack of data for some early-diverging members of the Viridiplantae (53) may complicate this analysis. Despite this problem and the relatively poor statistical support for this analysis, it is noteworthy that eukaryotic algal lineages other than dinoflagellates are each associated with a single GUN4 lineage, and dinoflagellate sequences are also recovered in distinct clades. Taken together, this analysis provides a framework for examining potential conservation of bilin binding in GUN4 proteins from different eukaryotic algae.

We therefore examined conservation of the LxNxLR motif in eukaryotic GUN4 proteins in this context. As expected, the LxNxLR motif is largely conserved in cyanobacterial GUN4 orthologs (Fig. 9). Apparent exceptions are proteins from *A. vandensis* and *A. panamensis*, lacking the initial Leu residue, and those of *G. lithophora* and related organisms, lacking the second Leu residue and sometimes exchanging Asp for Asn. The latter substitution may not prevent bilin binding, because the reverse Asp-to-Asn substitution in a conserved catalytic residue in PcyA does not preclude bilin association (54). All other cyanobacterial GUN4 orthologs retain the entire motif, including those from Thermostichales. By contrast, no member of the paralog clade retains the conserved Asn211/Asn219 (Fig. 1B), which we have established is essential for bilin binding in CrGUN4 (Fig. 8). Although one paralog sequence had the other three residues, all other paralogs lack at least two of the four residues within this motif. GUN4 paralogs from *Gloeobacter* spp. also lack these signature residues (Fig. 9). We therefore propose that the lack of this motif correlates with the loss of bilin binding in these cyanobacterial paralogs. In eukaryotes, the LxNxLR motif is well conserved in GUN4 proteins from glaucophyte and rhodophyte algae, which retain bilin-based phycobilisomes for light harvesting and hence also retain PCB (55, 56). A number of rhodophytes have other aliphatic residues in place of Leu213/Leu221, but Asn211/Asn219 is universally conserved in GUN4 from these algal lineages (Fig. 9). All four residues in this signature motif are conserved in GUN4 from Viridiplantae and from *Karenia* spp., consistent with the importance of bilin binding by GUN4 in Chlamydomonas (11). Some dinoflagellate GUN4 proteins retain the motif but have other aliphatic residues in place of Leu213/Leu221. This is similar to GUN4 proteins from rhodophytes, even though dinoflagellates no longer use phycobilisomes (57). There may also be a loose correlation between the sequence of this motif and the enzymes carrying out the final steps in bilin biosynthesis. GUN4 proteins from stramenopiles and most haptophytes retain most of the motif but have Ser in place of Asn211/Asn219, and such algae have either a PebA homolog, a PebB homolog, or both rather than the PcyA or HY2 enzymes that can synthesize PCB (58, 59). Ser is also present in place of Asn in cryptophyte GUN4 proteins. Cryptophytes retain diverged phycobiliproteins and possess PebA, PebB, and sometimes PcyA (58, 60). Overall, the LxNxLR motif is tightly conserved in photosynthetic eukaryotes with primary plastids (rhodophytes, glaucophytes, and Viridiplantae). Taken together with the known bilin binding by GUN4 from Synechocystis and Chlamydomonas, it seems likely that GUN4 from rhodophytes and glaucophytes will also bind PCB.

## Discussion

In this work, we have examined the evolution of GUN4 and of its potential for bilin binding. We have also identified additional metagenomic resources for early-diverging cyanobacterial lineages. Using phylogenetic analyses and characterization of recombinantly expressed proteins, we present evidence that GUN4 was present in LCCA and that bilin binding is an ancient feature of GUN4 orthologs that appears lost in paralogs. Our work thus presents several new insights.

### An extended understanding of early cyanobacterial evolution

Our analysis of 16S rDNA, of catenated ribosomal proteins, and of several proteins corroborates the recently proposed composition and phylogenetic analysis of Gloeobacterales (25), including assignment of “*A. lilacina*” (37) within *Gloeobacter* (Figs. 2, 3, S3–S5, and S14B). We also largely confirm the proposed position and composition of Thermostichales (27). We recover a well-supported clade including thermophilic isolates from hot springs in Yellowstone and Rupite, Bulgaria. This clade also contains multiple examples known to contain thylakoids (27, 43). In contrast to the original proposal for Thermostichales but in agreement with other recent studies (31, 61), *S. lividus* PCC 6715 groups with *Thermosynechococcus* spp. and *Synechococcus* sp. PCC 6312 in the higher crown cyanobacteria. We also identify at least one early-branching, mesophilic lineage within Thermostichales. This lineage is currently represented by three MAGs from South African stromatolites and by other 16S sequences (Fig. 2). The size of the two most complete assemblies assigned to this lineage, SM2_3_1 and SM2_3_2, is larger than complete genomes from hot springs isolates of the Thermostichales (Fig. 3B). This may indicate that the thermophilic members of this lineage have undergone some type of genome reduction, as has also been suggested for the thermophilic genus *Thermosynechococcus* (61). These studies provide a robust framework for examination of GUN4 origins, with a reproducible G/T/HC topology observed in analyses of proteins of varying size and of 16S rDNA sequences. We did not unambiguously assign two organisms to either Thermostichales or higher crown cyanobacteria, *Synechococcus* sp. PCC 7336 and SM2_3_60 (Fig. 4A), because of discrepancies between the 16S tree and ribosomal protein trees. At least one additional early cyanobacterial branch was potentially implicated in the 16S tree, and other potential early-diverging 16S sequences were omitted from our tree because of poor overlap with the 16S fragment from SpSt-379. Hence, it seems likely that this G/T/HC topology may need to be amended or expanded as additional data become available.

### An early origin for GUN4

Unlike MgCh, GUN4 has no ortholog in anoxygenic photosynthetic organisms. Hence, there is no rigorous outgroup available. As an alternative approach, we compared phylogenetic analysis of GUN4 with that of the G/T/HC topology we observed for early-branching cyanobacteria, which is itself in good agreement with a range of previous studies (Fig. S1; (21, 23), 25–31, 33–35)). This comparison reveals that the observed topology of GUN4 orthologs matches that expected for an ancestral cyanobacterial protein. GUN4 paralogs do not exhibit such a topology, implying that the ancestral cyanobacterial GUN4 ortholog was specifically lost in *Gloeobacter* spp. Alternatively, one could instead assume that GUN4 paralogs gave rise to orthologs late in cyanobacterial evolution. In our tree, the GUN4 orthologs most closely related to paralogs are found in *G. lithophora* and related crown cyanobacterial species. Hence, subsequent HGT events must be invoked to account for the presence of GUN4 orthologs in earlier branches such as Thermostichales. Moreover, GUN4 orthologs within Thermostichales form a robust, well-supported clade with a basal mesophilic sequence. This is consistent with the presence of a GUN4 ortholog in the common ancestor of all Thermostichales, implying that this organism diverged from other cyanobacteria and then remained a single genetic population until GUN4 orthologs had evolved elsewhere and were introduced by HGT.

This hypothetical late evolution of GUN4 from paralogs would be placed at approximately the same time as establishment of primary plastid endosymbiosis, because GUN4 from *G. lithophora* and related organisms are the closest cyanobacterial orthologs both to the cyanobacterial GUN4 paralogs (Figs. 5B and S15–S18) and to the presumptive GUN4 orthologs from glaucophyte and rhodophyte algae (Figs. 9 and S19). Thermostichales would thus have to have remained a single population from the time of their initial divergence until approximately the time of plastid establishment. Molecular clock estimates place branching of Gloeobacterales from crown cyanobacteria at 2.5–2.9 Gya (26, 62, 63). Studies including Thermostichales as a separate branch indicate their emergence relatively shortly thereafter: one analysis places branching of Gloeobacterales at 2.9 Gya and that of Thermostichales at 2.8 Gya (62), whereas a more recent analysis favored slightly more recent appearances of 2.6 Gya and 2.5 Gya, respectively (63). The subsequent appearance of Pseudanabaena at 2.6 or 2.3 Gya in the same reports (62, 63) is consistent with the presence of filamentous cyanobacterial microfossils in formations dated at ca. 1.9 Gya (64, 65). Plastid endosymbiosis is thought to have occurred more recently, with estimates ranging from 1.6–1.8 Gya in some studies to 2.1 Gya or 0.9 Gya in others (63, 66–68). Hence, the most conservative estimate requires Thermostichales to have branched from other cyanobacteria at 2.5 Gya and acquired a GUN4 ortholog no earlier than 2.1 Gya.

These estimates allow a minimum time of 400 million years during which Thermostichales remained a single genetic population such that a single HGT event would have given rise to vertically inherited GUN4 in this lineage. Cyanobacteria are certainly capable of retaining morphology and cell division patterns for billions of years (65), but it seems implausible that the ancestors of Thermostichales would have remained a single genetic population for this time without geographic dispersal or diversification into thermophilic niches. Moreover, there is no corroborating evidence that the GUN4 binding partner CHLH was similarly acquired via HGT in Thermostichales. We hence favor the simpler explanation of these observations: GUN4 orthologs were present in LCCA and were lost in Gloeobacter spp. This scenario is consistent with the hypothesis that GUN4 is an ancient and essential component in the process of adapting MgCh for function in the presence of oxygen.

### A widely conserved bilin-binding motif

We have also demonstrated that a point substitution is sufficient to ablate binding of bilin but not porphyrin in CrGUN4. Retention of porphyrin binding also rules out an overall loss of structural integrity, implicating this N219G substitution as directly affecting ligand specificity. Asn219 is part of an LxNxLR motif found in both CrGUN4 and SyGUN4, and the equivalent Asn211 residue is in close proximity to bound bilin in crystal structures of SyGUN4 (Fig. 1B; (2)). We therefore used the diversification of this motif in eukaryotic GUN4 sequences to assess the potential distribution of bilin binding within different algal lineages. The results of this analysis provide four insights. First, three out of four residues in this motif are conserved in GUN4 orthologs from Gloeobacterales, including the critical Asn residue, implying that bilin binding is likely to have arisen early in GUN4 evolution. Second, the motif is largely conserved in cyanobacterial GUN4 orthologs but not in paralogs, implying a correlation between bilin binding and GUN4 function. Third, the motif is conserved in orthologs from glaucophyte and rhodophyte algae, Viridiplantae, and most dinoflagellates, implying that bilin binding by GUN4 is conserved in these organisms. Fourth, eukaryotic GUN4 sequences lacking an equivalent to Asn219 instead have Ser at this position. Such haptophytes, cryptophytes, and stramenopiles have secondary plastids derived from rhodophyte algae and do not have the PCYA or HY2 enzymes needed to synthesize PCB. The substitution of Ser for Asn may thus reflect an adaptation to binding a different, physiologically relevant bilin in these organisms. It will be interesting to test this idea via reconstitution of FDBR activity from such organisms and via characterization of their GUN4 proteins, although the poor chromophorylation of Synechocystis GUN4 upon heterologous expression (2) and the absence of known assays for function of GUN4 paralogs indicates that improved assays are likely needed for such studies. Similar studies on GUN4 from Gloeobacterales and Thermostichales would also test our proposal that bilin-binding GUN4 orthologs were an ancient component essential for Chl biosynthesis in an oxic environment in the LCCA.

## Materials and methods

### Phylogenetic analyses

Metagenomic and genomic assemblies from Gloeobacterales and Thermostichales (20, 21, 24, 25, 32, 36, 37, 39, 42) were downloaded for use as local BLAST (19) databases. The same approach was used for *Synechococcus* sp. PCC 7336, SM2_3_60, *Synechococcus* sp. C9 (69), and recently deposited MAGs for members of the Acaryochloridaceae and Gloeomargaritales (28, 32, 70) and for a microbial mat community from Shark Bay, Australia (71). Multiple sequence alignments were constructed using MAFFT v7.450 (72). Maximum likelihood phylogenies were inferred using PhyML 3.1 (73), and statistical robustness was assessed using the transfer bootstrap expectation (TBE) as calculated in booster v0.1.2 (74). For Bayesian analysis, the alignment of cyanobacterial GUN4 proteins was converted into NEXUS format for use in MrBayes v3.2.7a (75) using the command-line -convert feature in CLUSTAL (76). Additional details are reported in the Supplemental Text.

### Characterization of recombinant GUN4 proteins

A synthetic gene encoding N219G CrGUN4 was obtained from GenScript as in-frame His-tagged fusion proteins in pET28a, matching the previously reported expression construct for wild-type (11). Wild-type and N219G CrGUN4 were expressed in *E. coli* C41 (77) with plasmid pKT271 for PCB biosynthesis (48) and then purified from frozen cell pellets as described (11). Briefly, frozen cell pellets were thawed and resuspended prior to lysis via repeated passage through a microfluidizer. Lysate was clarified by centrifugation, and protein was then bound to Talon Ni^2+^–NTA resin (Novagen) and eluted using an imidazole gradient. Peak fractions were pooled for overnight dialysis against TKKG buffer (25 mM TES-KOH pH 7.8, 100 mM KCl, 10% (*v*/*v*) glycerol) at 4°C. Dialyzed protein samples were then analyzed using a Cary 60 spectrophotometer. Fluorescence spectra were acquired using a QM-6/2005SE fluorimeter with red-enhanced photomultiplier tubes (Photon Technology International 814 Series).

### Data accessibility

Text files for absorption and fluorescence spectra and for each phylogenetic analysis generated during the current study are available at https://doi.org/10.25338/B8505X via the DataDryad repository.

## Supplementary Material

pgad131_Supplementary_Data
