# Supplementary material for: GUN4 appeared early in cyanobacterial evolution
Source: PNAS Nexus. 2023 Apr 12;2(5):pgad131. doi: 10.1093/pnasnexus/pgad131 (PMC10156173; doi:10.1093/pnasnexus/pgad131)
Supplement: pgad131_Supplementary_Data [file pgad131_supplementary_data.pdf]

PNAS nexus

Supplementary Information for GUN4 appeared early in cyanobacterial evolution

Nathan C. Rockwell* and J. Clark Lagarias*

* Nathan C. Rockwell, J. Clark Lagarias

Email: ncrockwell@ucdavis.edu jclagarias@ucdavis.edu

This PDF file includes:

Supplementary text
Figures S1 to S19
Tables S1 to S2
SI References

Supplementary Information Text

Methods.

Phylogenetic analyses. Metagenomic and genomic assemblies from Gloeobacterales and Thermostichales (20, 21, 24, 25, 32, 36, 37, 39, 42) were downloaded for use as local BLAST (19) databases. The same approach was used for *Synechococcus* sp. PCC 7336, SM2_3_60, *Synechococcus* sp. C9 (69), and recently deposited MAGs for members of the Acaryochloridaceae and Gloeomargaritales (28, 32) and for a microbial mat community from Shark Bay, Australia (70). BLAST searches against NCBI and DOE-IMG databases were used to identify other sequences. Construction of catenated alignments of ribosomal proteins is described in the Supplemental Text. MAFFT v7.450 (71) was used to construct multiple sequence alignments (command-line settings --genafpair --maxiterate 16 --clustalout –reorder). The resulting alignments were screened for sequences at ≥ 98% identity. Several groups often fell within that limit for the proteins and assemblies in this study: *A. vandensis* LV9 and MP9P1; *G. violaceus*, *G. morelensis*, and "*A. lilacina*;" *Synechococcus* spp. M44_DOE_062, SpSt-164, and SpSt-285; and *Synechococcus* spp. JA-3-3Ab, 60AY4M2, 63AY4M1, 63AY4M2, 65AY640, 65AY6A5, and 65AY6Li (Fig. 4A). Redundant examples of such sequences were omitted from final protein phylogenies, and gap-enriched columns (≥5%) were removed using an in-house script which also generated input files for PhyML 3.1 (72). The alnfilter utility, distributed with homolmapper (73), was used to check for sequences with <90% of remaining characters; such sequences were removed from analysis except for selected 16S sequences and one GUN4 paralog from *Pseudanabaena*. Retained incomplete sequences are indicated in the figures, and all alignments were recalculated after removal of incomplete sequences. Inclusion of both GUN4 orthologs and CHLH proteins from *Pseudanabaena* spp. in some alignments resulted in a substantial change in the number of well-aligned positions (defined as ≤5% gaps). GUN4 orthologs from *Pseudanabaena* were therefore analyzed separately, along with a GUN4 sequence from a MAG reported from a pustular microbial mat in Shark Bay, Australia (70) and with removal of a paralog sequence that was aligned poorly when *Pseudanabaena* orthologs were present. Maximum-likelihood phylogenies were inferred using PhyML 3.1 with 100 bootstraps. Command-line settings for nucleic acid alignments were -m GTR -s SPR -a e -c 4 -v e -o tlr -b 100, and command-line settings for protein alignments were -m WAG -d aa -s SPR -a e -c 4 -v e -o tlr -b 100. Statistical robustness was assessed using the transfer bootstrap expectation (TBE) as calculated in booster v0.1.2 (74). For Bayesian analysis, the alignment of cyanobacterial GUN4 proteins was converted into NEXUS format for use in MrBayes v3.2.7a (75) using the command-line -convert feature in CLUSTAL (76). The resulting file was used to infer a Bayesian phylogeny with 3,000,000 generations and other settings as defaults (4 chains for each of 2 runs, temperature factor of 0.1, and a burn-in fraction of 25%). For the alignment of cyanobacterial and algal GUN4 proteins, the Bayesian phylogeny was inferred with 1,750,000 generations using 6 chains for each of 2 runs, a temperature factor of 0.05, and the same burn-in fraction. The final average standard deviation of split frequencies was below 0.1 in both cases.

Construction of catenated ribosomal proteins. We used annotated ribosomal proteins from the genome of *Thermosynechococcus elongatus* BP-1 (1) as queries in pilot BLAST (2) searches. These searches indicated that approximately 50% of the shorter ribosomal proteins in this group were missing in the MAG for Gloeobacterales sp. SpSt-379 (3, 4). We therefore used *T. elongatus* ribosomal proteins of ≥ 128 amino acids as queries, which gave a set of 23 query proteins (Table 2). This criterion gave approximately 70% coverage in the SpSt-379 MAG and ≥85% coverage for SM2_3_1 and SM2_3_2, assigned as mesophilic members of the Thermostichales, with a detailed comparison of those MAGs to *T. elongatus* presented in Table S2. An in-house Python script was used to generate a spreadsheet containing the best BLAST hit for each query in a given protein assembly. Two other MAGs were identified as possible close relatives of SM2_3_1 and SM2_3_2. One of these, RM1_1_27, was very incomplete, containing only four proteins from this set (Table S2). Three of the four were identical to proteins found in SM2_3_2, so RM1_1_27 was not studied further. The other MAG, SM2_3_60, had only ten proteins in this set, one of which was a fragment. The proteins present in SM2_3_60 had little overlap with those present in SpSt-379 (Table S2). We therefore constructed two catenations. One catenation was based on good overlap of SpSt-379 with SM2_3_1, SM2_3_2, and other genomes and MAGs from Gloeobacterales and Thermostichales. This comprised eight proteins: S5, L15, L13, S9, S4, L1, S7, and L9, in that order. The second catenation was based on good overlap of SM2_3_60 with the same genomes and MAGs. This comprised four proteins: L16, L5, S8, and L1, in that order. Three

proteins present in SM2_3_60 were omitted due to their absence in SM2_3_1 or SM2_3_2 (Table S2) or in *Aurora vandensis* (ribosomal protein L15). A spreadsheet containing a list of targets for each assembly was then constructed for each catenation, and an in-house Python script was used to extract the protein sequences from each assembly and reorder them. Sequences from *Aurora vandensis* and from the Arctic peat metagenome were added manually, because available assemblies are for nucleic acid sequence and needed to be translated. Sequences from *Cyanothece* sp. PCC 7425 were also added manually, because the only available assembly was from DOE-IMG and contained different header information than NCBI assemblies. The resulting catenated amino acid sequences were then aligned using MAFFT v7.450 (5) and used to infer maximum likelihood phylogenies with PhyML-3.1 (6) as described above. Statistics for the resulting trees are presented in Table S1.

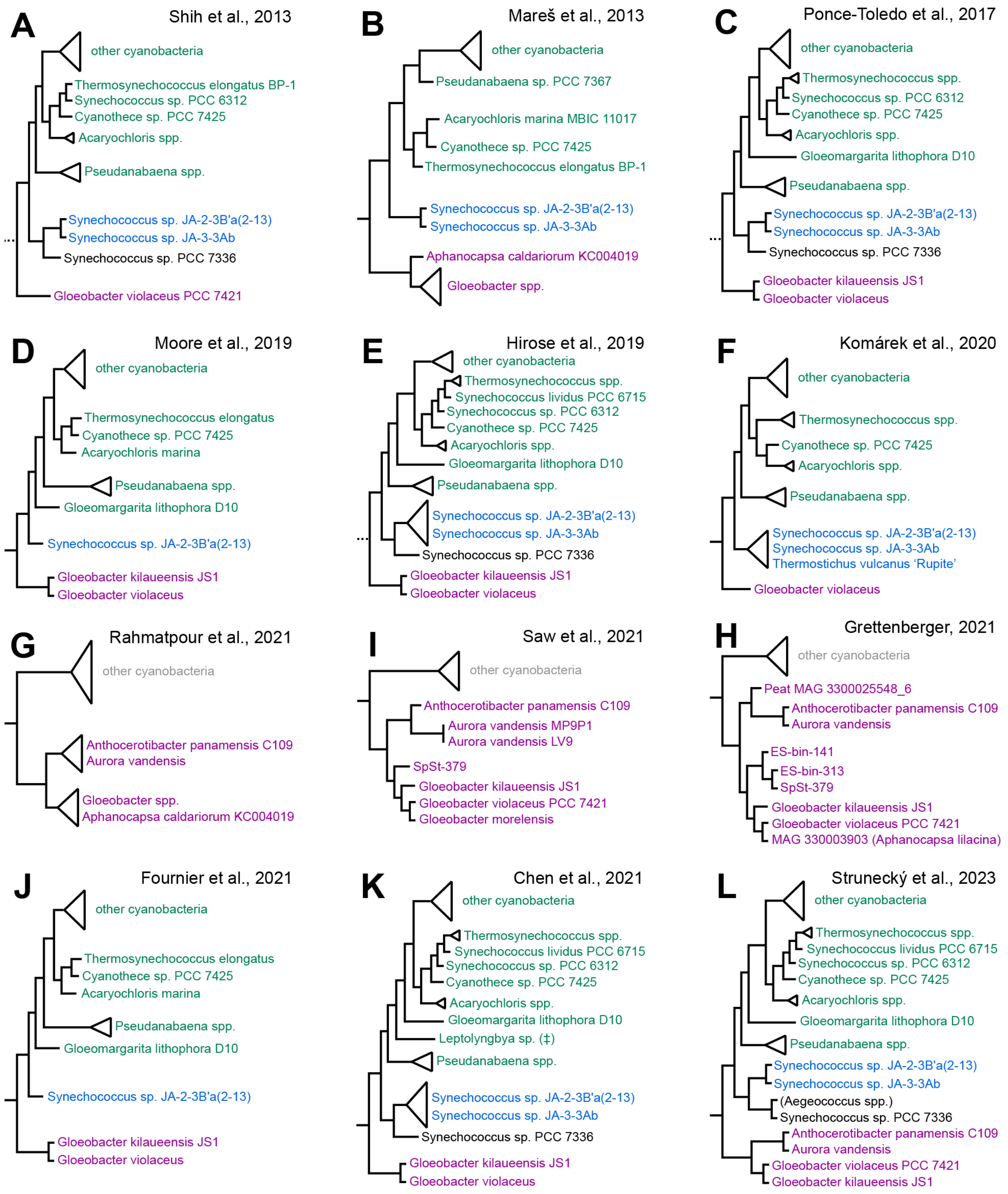

Fig. S1. Phylogenetic analyses of early-branching cyanobacterial lineages. The topology of early branches in cyanobacterial evolution is shown for studies over the past decade (3, 4, 7-16) using the color scheme of Fig. 2 (Gloeobacterales, mauve; Thermostichales, blue; higher crown cyanobacteria, green). Cases in which *Gloeobacter* spp. were used for root placement without a non-cyanobacterial outgroup (panels A, C, and E) have dashed roots. In panels E, F, and K, triangles for Thermostichales include the listed taxa but also include other taxa or sequences. Genomes have not yet been reported for *Aegeococcus* (panel L, (17)). One study (panel K, (13)) reported a potential additional early-branching isolate within higher crown cyanobacteria assigned to *Leptolyngbya* (‡).We did not analyze sequences from this organism due to discrepancies in the strain numbers between the publication and the deposited assemblies, which prevented us from identifying the assembly in question.

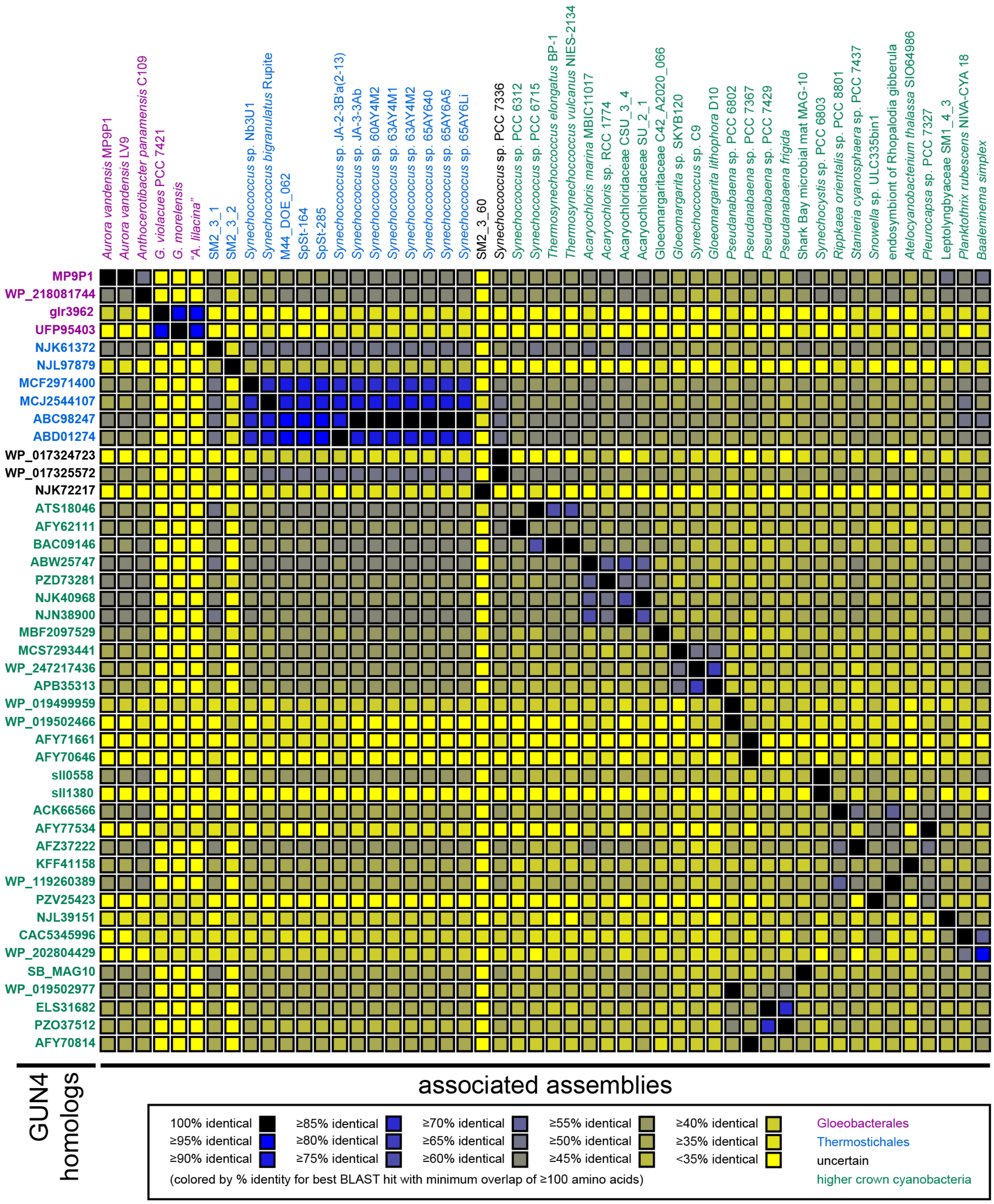

Fig. S2. Relative conservation of cyanobacterial GUN4 homologs. Proteins used for phylogenetic analysis of cyanobacterial GUN4 homologs (Figs. 5, 6, S15–S17) were used as queries in BLAST searches against the assemblies containing those proteins. The results are shown colored by percent identity for regions with ≥100 amino acids. In this representation, distant paralogs give rise to bright yellow rows (as for glr3962), and assemblies lacking GUN4 orthologs give rise to bright yellow columns (as for *Gloeobacter violaceus* PCC 7421).

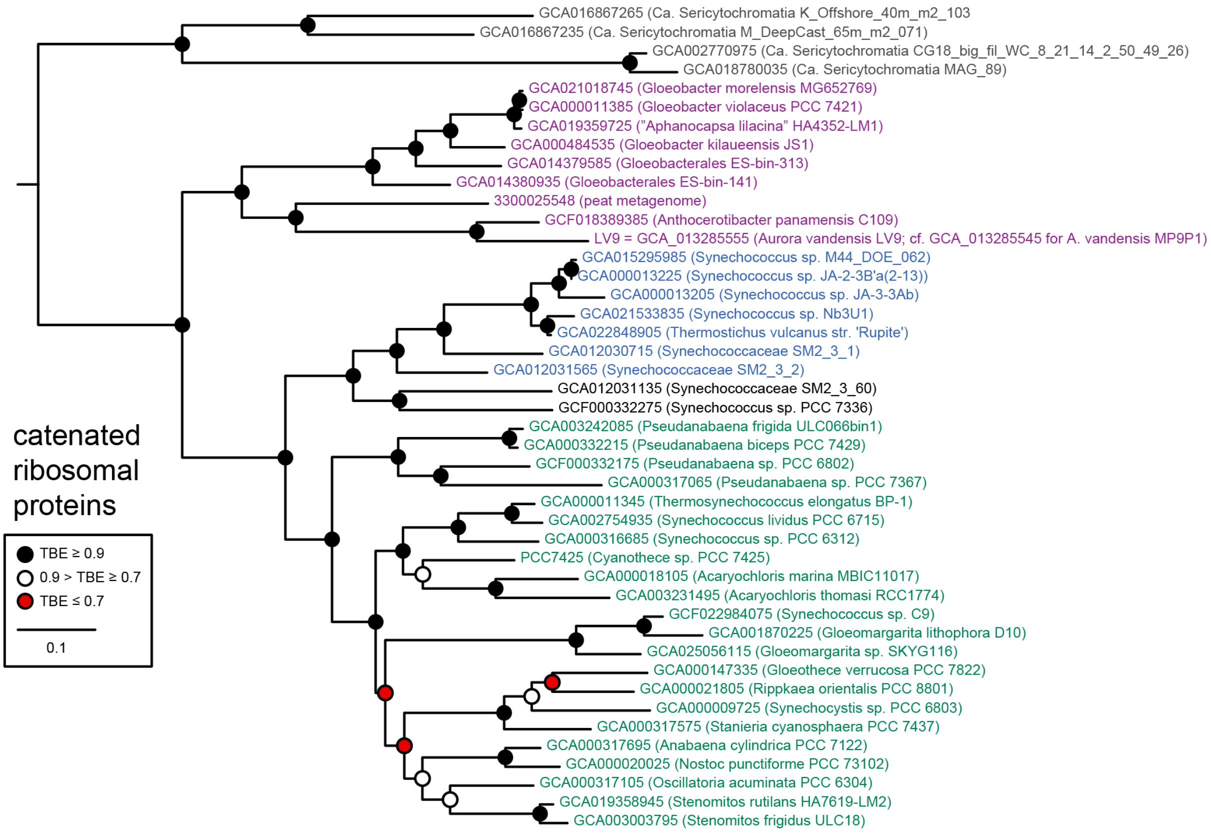

Fig. S3. Additional phylogenetic analysis of catenated ribosomal proteins. A catenation of ribosomal proteins was constructed and used to infer a maximum likelihood phylogeny for placement of MAG SM2_3_60. The resulting phylogeny is shown in the color scheme of Fig. 2, with the assemblies used for each organism indicated. This catenation comprised four proteins: L16, L5, S8, and L1, in that order. The root is placed between the outgroup (Sericytochromatia sequences) and all cyanobacteria.

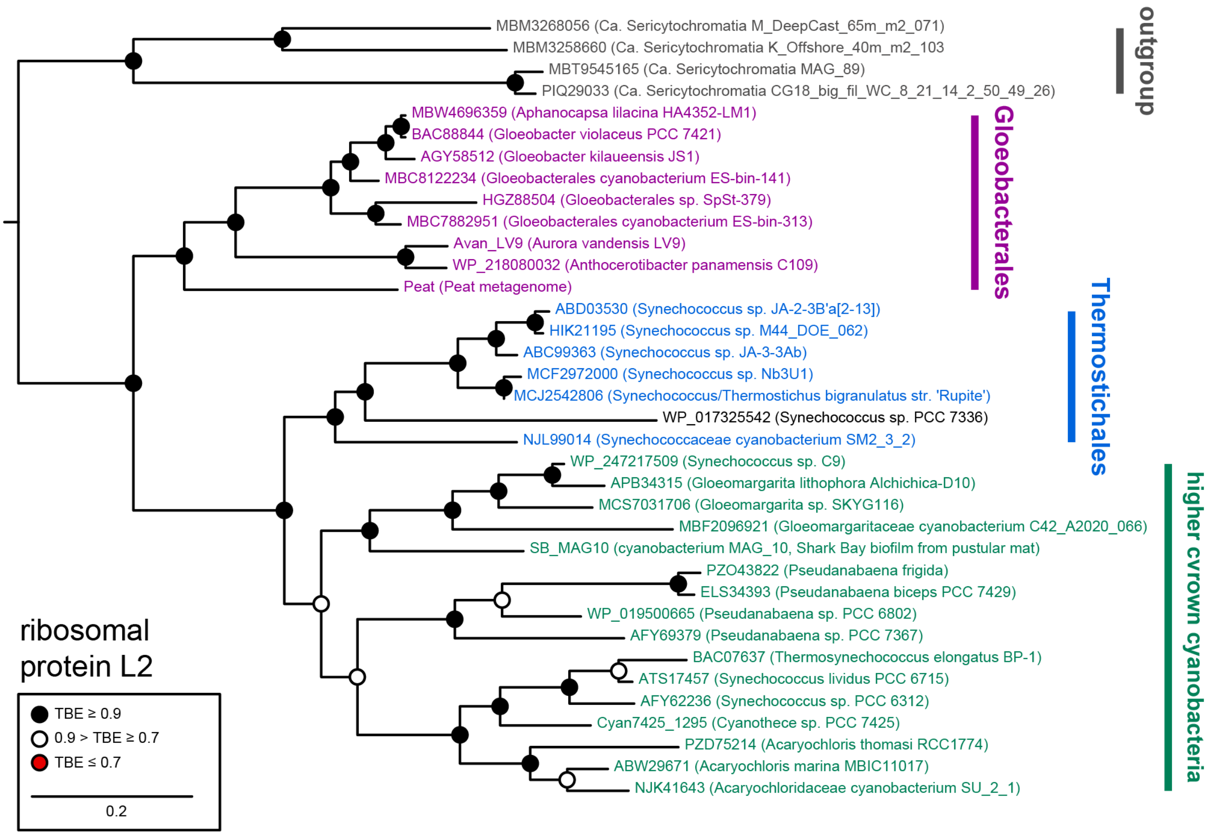

Fig. S4. Phylogenetic analysis of ribosomal protein L2. A maximum-likelihood phylogenetic tree is shown for ribosomal protein L2, using the color conventions of Fig. 2. Root placement is between L2 protein from cyanobacteria and all other bacteria. L2 was not part of the catenations shown in Fig. 3 and Fig. S3 (see Supplemental Text). The G/T/HC topology is indicated.

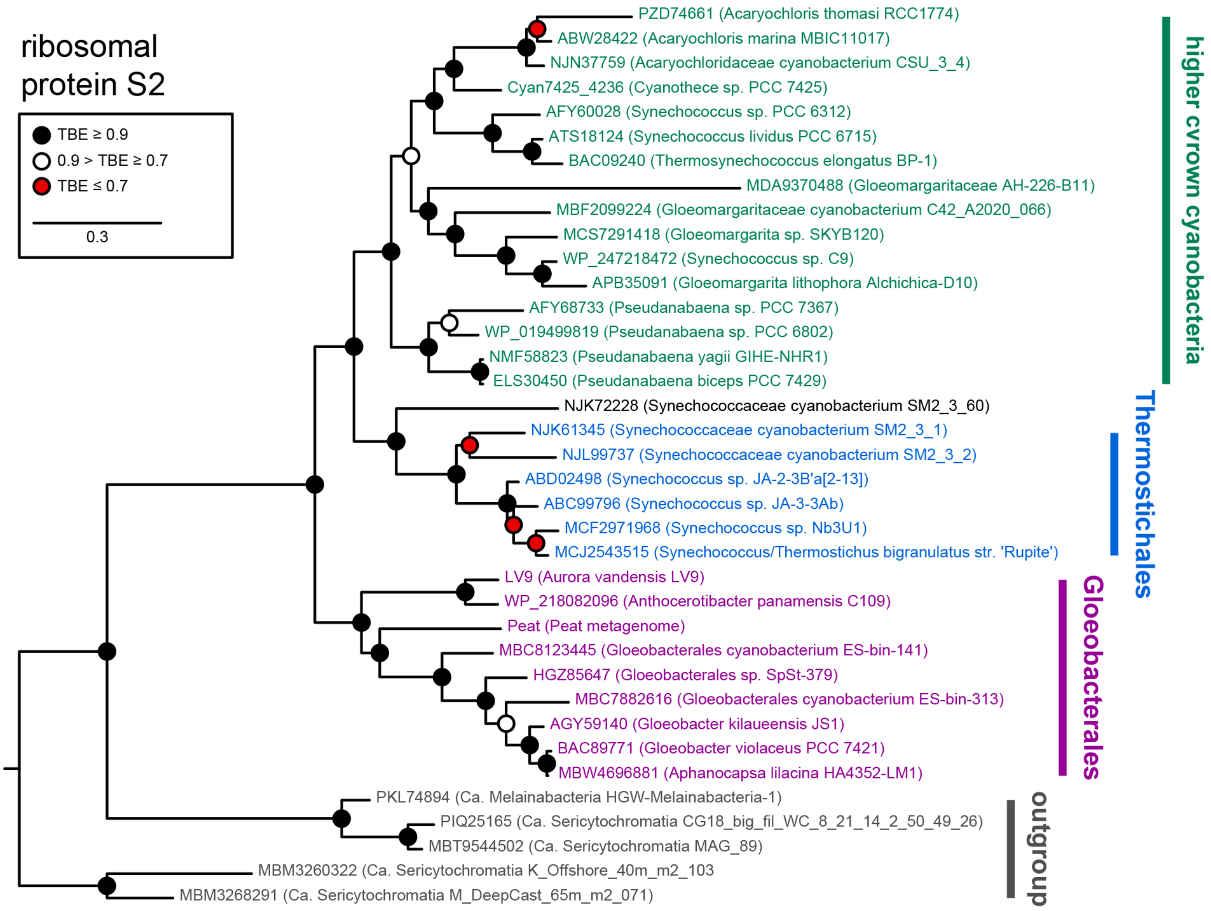

Fig. S5. Phylogenetic analysis of ribosomal protein S2. A maximum-likelihood phylogenetic tree is shown for ribosomal protein S2, using the color conventions of Fig. 2. Root placement is between S2 protein from cyanobacteria and all other bacteria. S2 was not part of the catenations shown in Fig. 3 and Fig. S3 (see Supplemental Text). The G/T/HC topology is indicated.

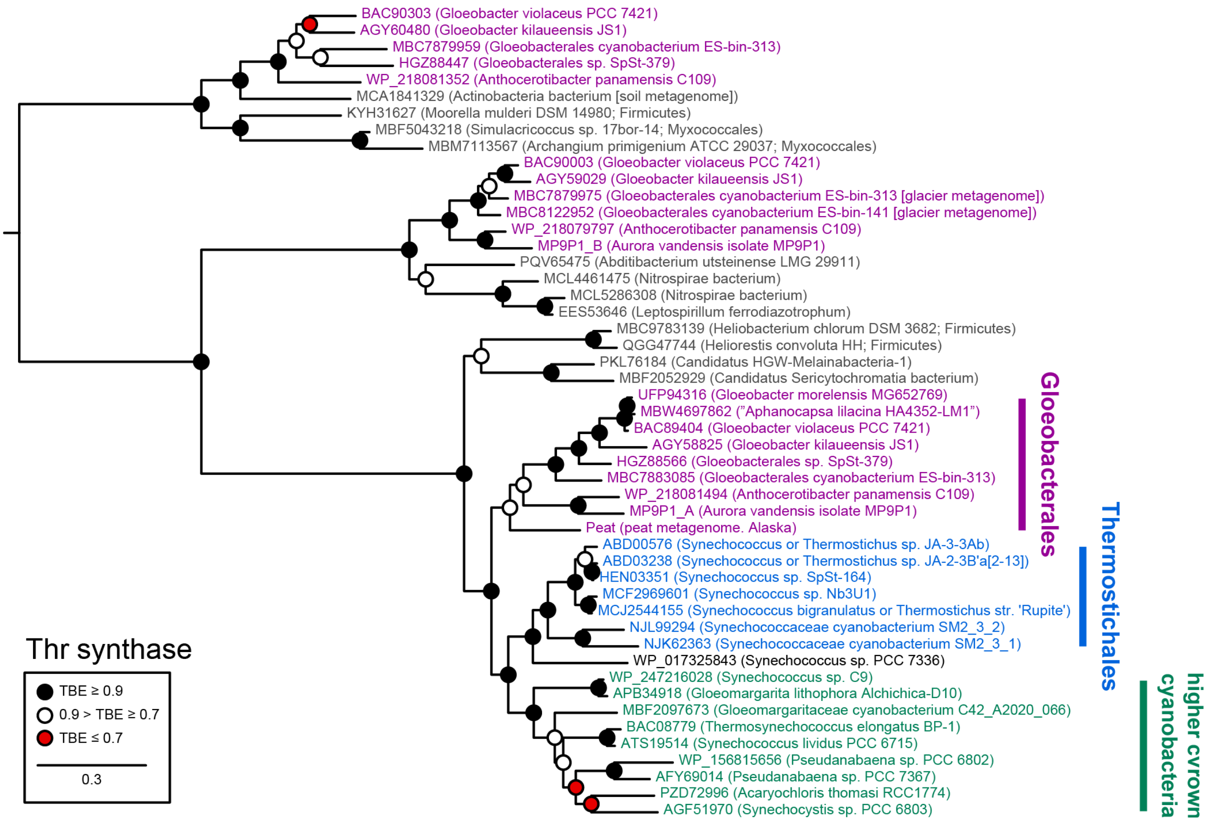

Fig. S6. Phylogenetic analysis of Thr synthases. A maximum-likelihood phylogenetic tree is shown for Thr synthase proteins, using the color conventions of Fig. 2. At least three Thr synthase sequences could be identified in the genome of *G. violaceus*, but the clade containing HGZ88566 was chosen for detailed analysis. Root placement is between the most distantly related clade and the other two, as judged by branch length, and the G/T/HC topology is indicated.

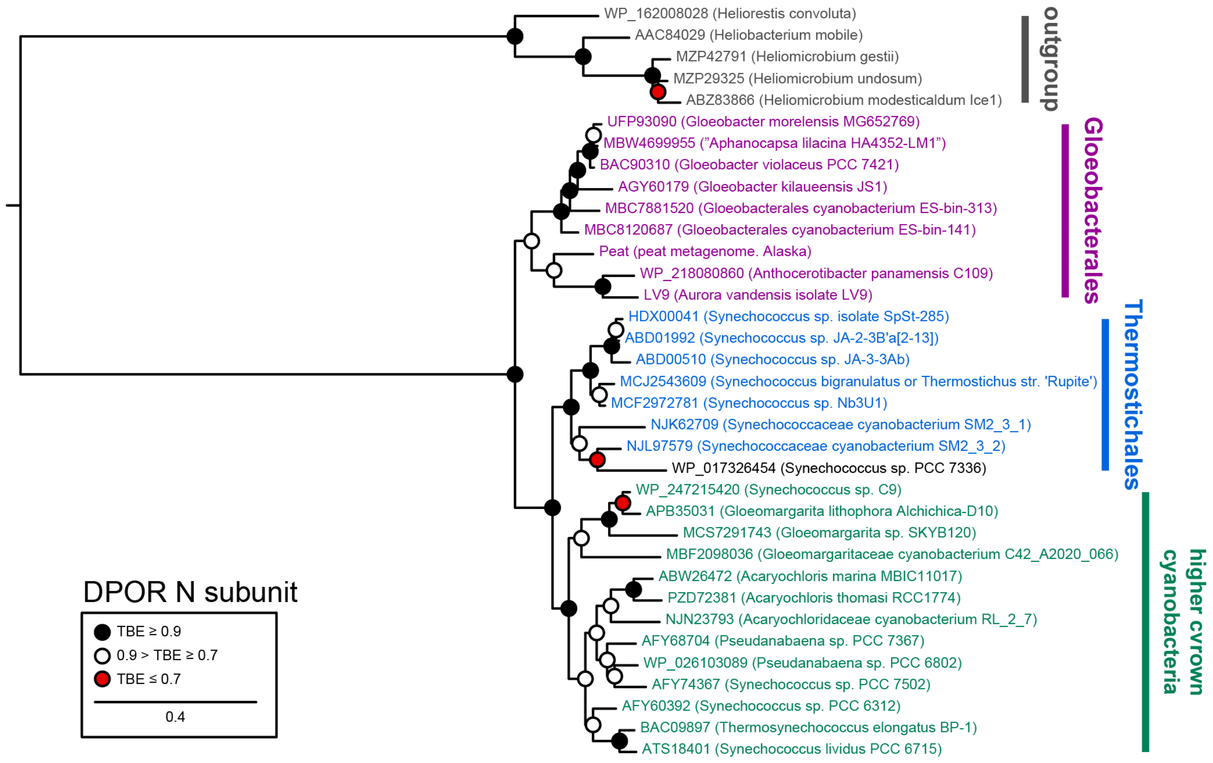

Fig. S7. Phylogenetic analysis of the N subunit of dark-operative protochlorophyllide oxidoreductase (DPOR). A maximum-likelihood phylogenetic tree is shown for the N subunit of DPOR, using the color conventions of Fig. 2. Root placement is between DPOR N subunits from Heliobacteria and those from cyanobacteria. The G/T/HC topology is indicated.

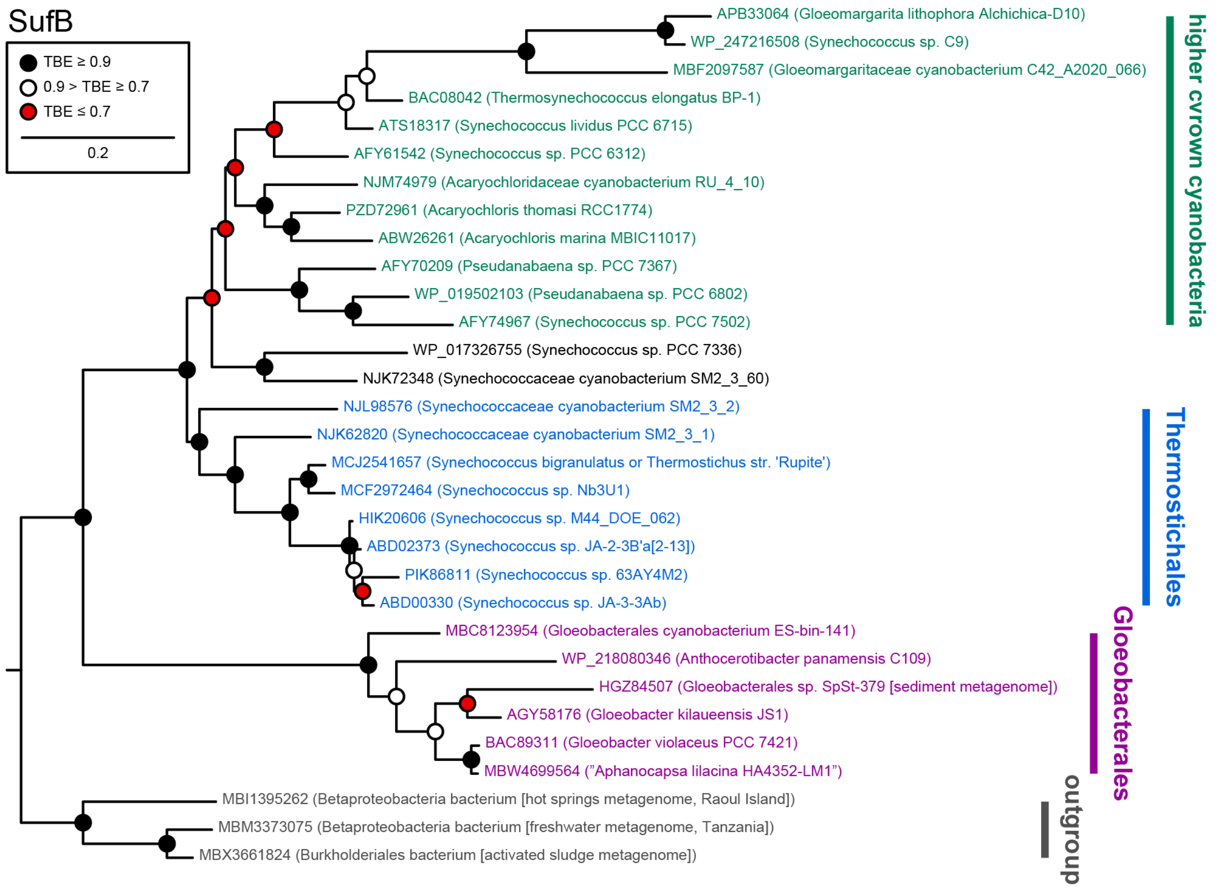

Fig. S8. Phylogenetic analysis of SufB. A maximum-likelihood phylogenetic tree is shown for SufB protein, using the color conventions of Fig. 2. Root placement is between SufB from cyanobacteria and all other bacteria. The G/T/HC topology is indicated.

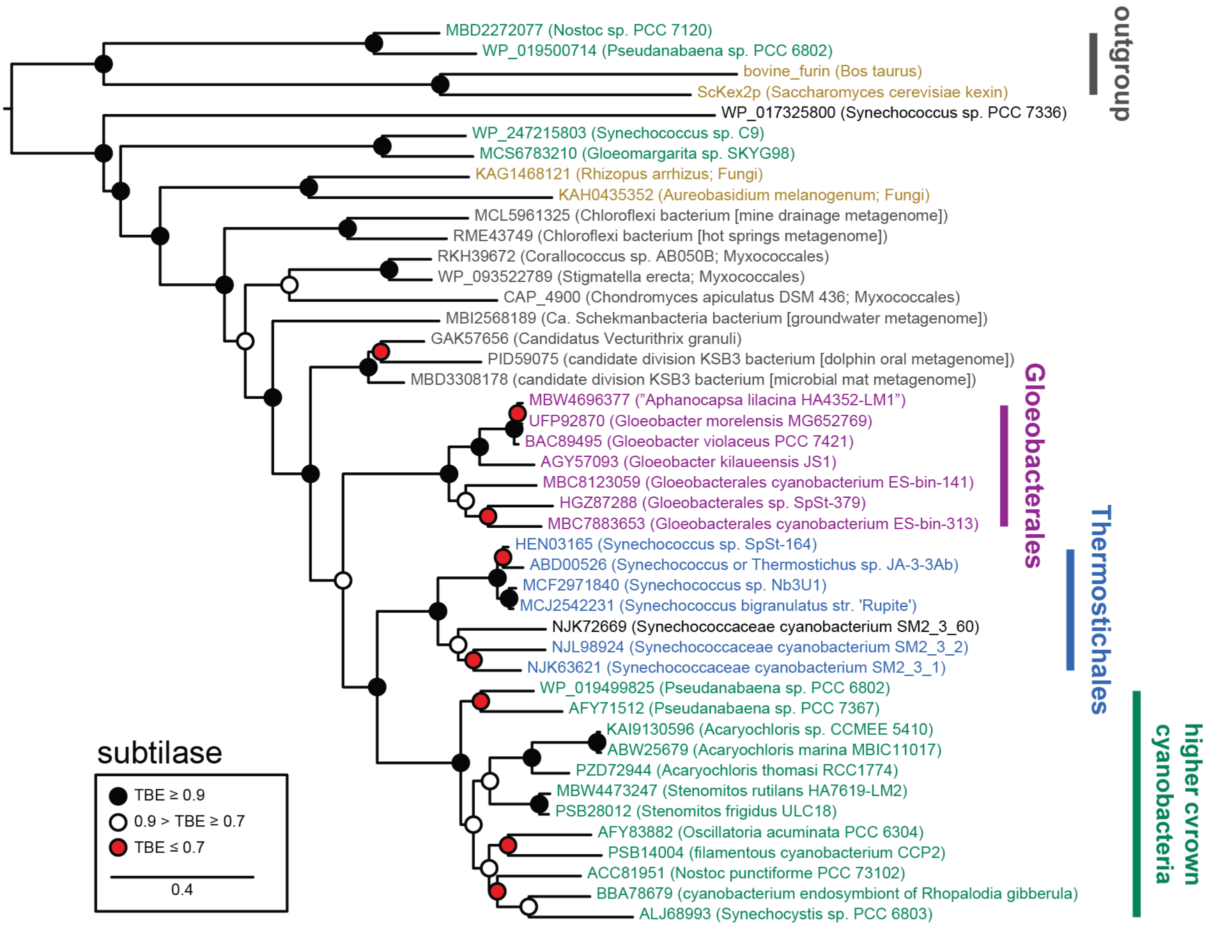

Fig. S9. Phylogenetic analysis of subtilases. A maximum-likelihood phylogenetic tree is shown for subtilases, using the color conventions of Fig. 2 with eukaryotic sequences indicated in ochre. Root placement is between a protease clade including proprotein convertases (18) and all other proteases. The G/T/HC topology is indicated.

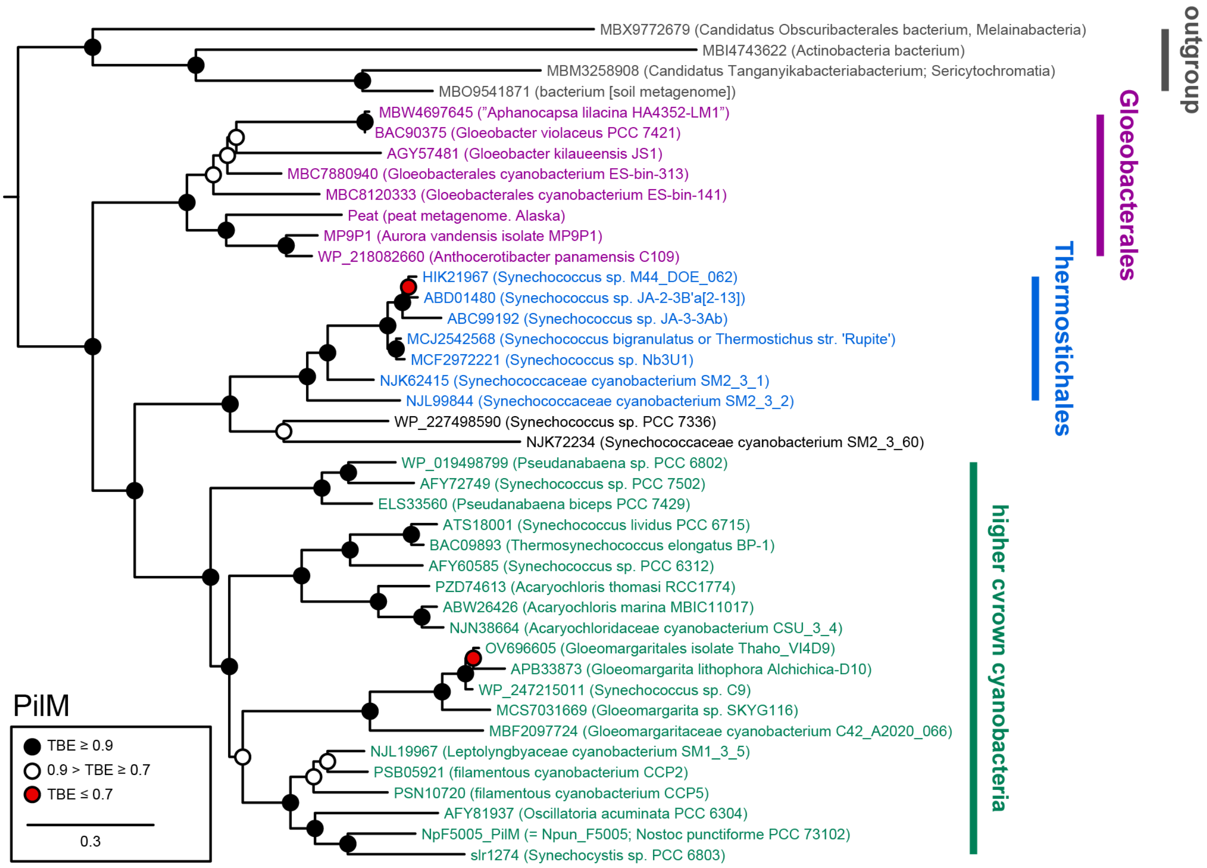

Fig. S10. Phylogenetic analysis of PilM. A maximum-likelihood phylogenetic tree is shown for PilM, using the color conventions of Fig. 2. Root placement is between PilM from cyanobacteria and all other bacteria. The G/T/HC topology is indicated.

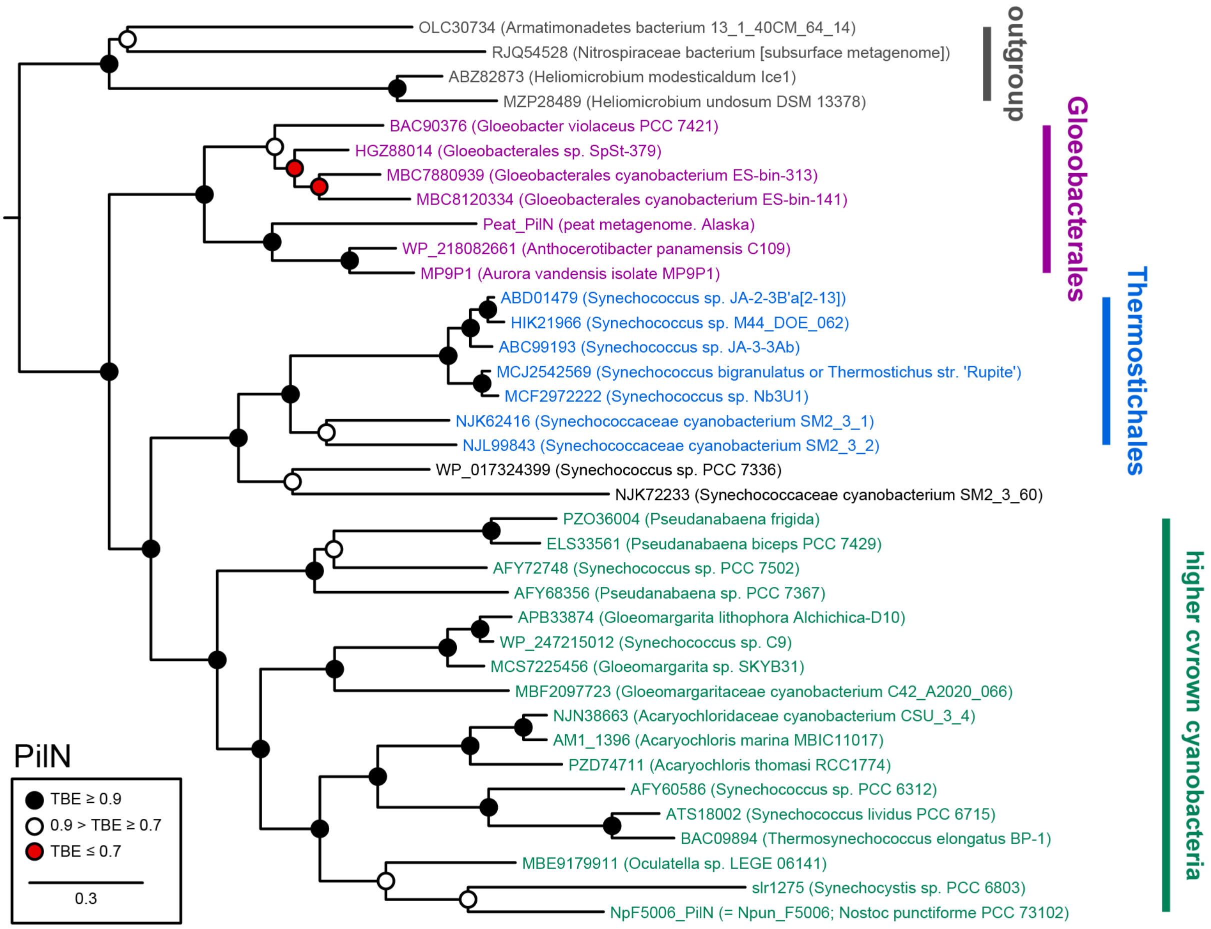

Fig. S11. Phylogenetic analysis of PilN. A maximum-likelihood phylogenetic tree is shown for PilN, using the color conventions of Fig. 2. Root placement is between PilN from cyanobacteria and all other bacteria. The G/T/HC topology is indicated.

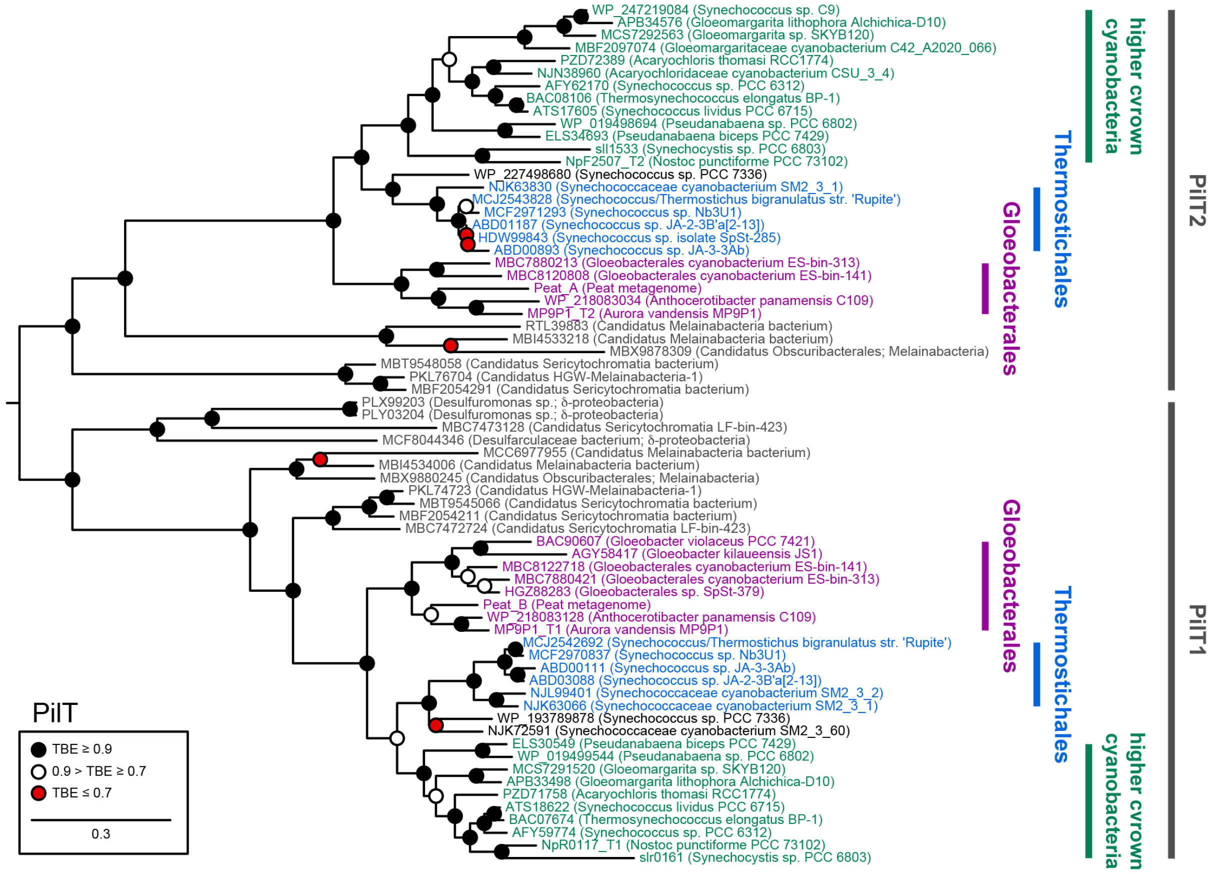

Fig. S12. Phylogenetic analysis of PilT proteins. A maximum-likelihood phylogenetic tree is shown for PilT proteins, using the color conventions of Fig. 2. PilT1 and PilT2 isoforms were assigned based on analysis of these proteins in *Nostoc punctiforme* PCC 73102 (19). Root placement is between PilT1 and PilT2. Non-cyanobacterial sequences were included for both isoforms. The G/T/HC topology was recovered for both PilT1 and PilT2, as indicated.

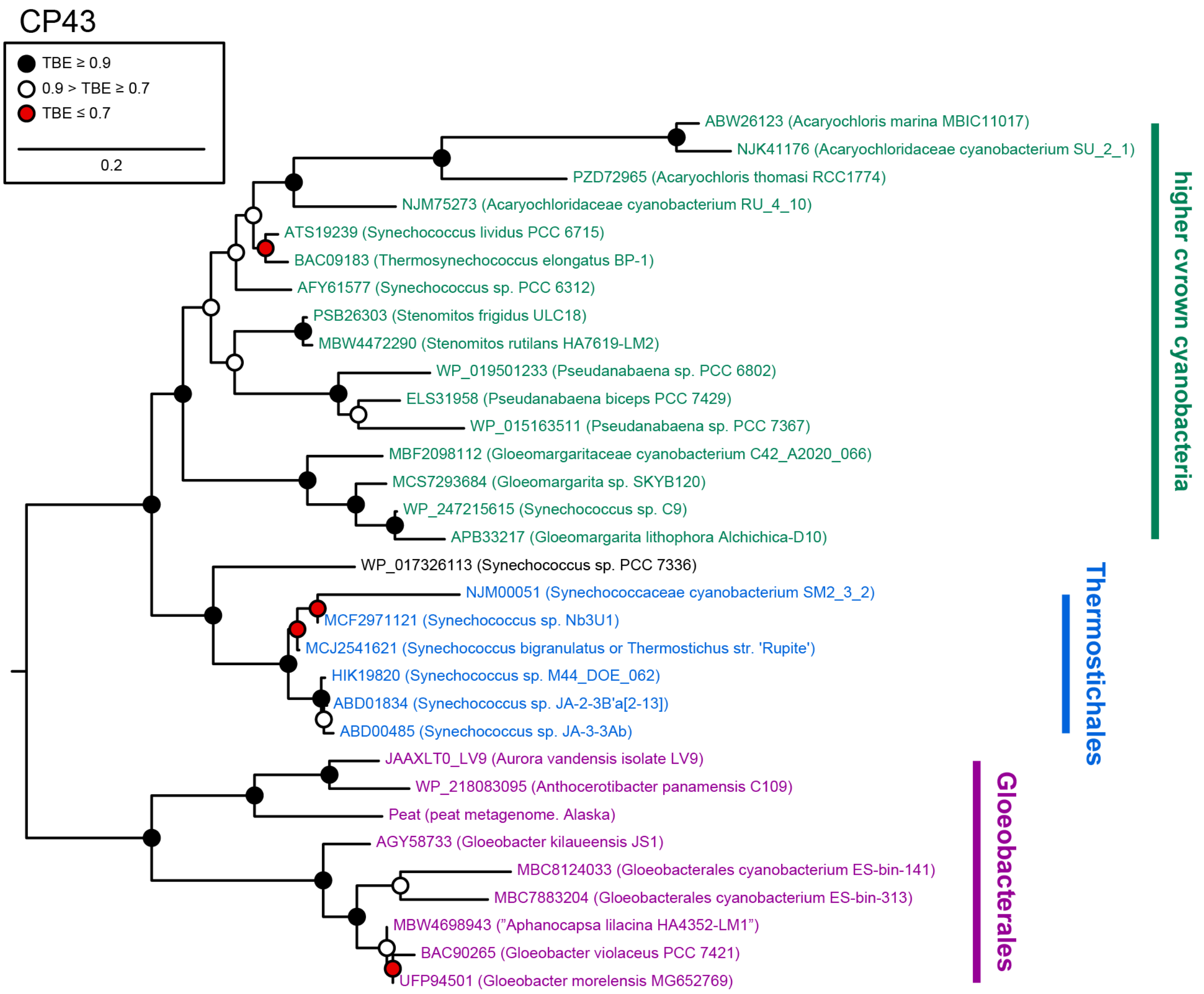

Fig. S13. Phylogenetic analysis of CP43. A maximum-likelihood phylogenetic tree is shown for CP43, using the color conventions of Fig. 2. Like GUN4, CP43 is confined to oxygenic photosynthetic organisms. Root placement is between CP43 from Gloeobacterales and all other bacteria. The G/T/HC topology is indicated.

A

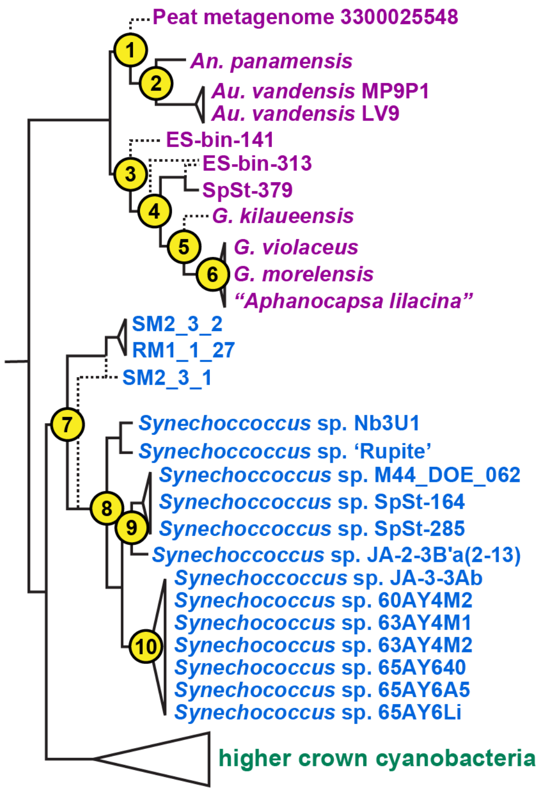

B

	ribosomal protein L2	ribosomal protein S2	Thr synthase	DPOR N	SufB	PilM	PilN	PilT1	PilT2	CP43	subtilase	support
1	N	N	N	Y	Ø	Y	Y	Y	Y	Y	Ø	6/9
2	Y	Y	Y	Y	Ø	Y	Y	Y	Y	Y	Ø	9/9
3	N	Y	Ø	Y	N	Y	N	N	Y	N	Y	5/10
4	Y	N	N	Ø	Ø	Ø	N	Y	Ø	Ø	Y	3/6
5	Y	Y	Y	Y	N	Y	Ø	N	Ø	N	Y	6/9
6	Y	Y	Y	Y	Y	Y	Y	Y	Ø	Y	Y	10/10
7	Y	Y	Y	Y	N	N	Y	Y	Y	N	Y	8/11
8	Y	N	Y	Y	Y	Y	Y	Y	Y	N	Y	9/11
9	Y	Y	Y	Y	N	Y	Y	Y	Y	N	Y	9/11
10	Y	Y	Y	Y	Y	Y	Y	Y	Y	N	Y	10/11

Fig. S14. Recovery of the G/T/HC topology using single-protein trees. (A) Single-gene trees (Figs. S4-S13) were used to construct an approximate topology for early-diverging cyanobacterial lineages. (B) Support at each of ten nodes was analyzed in each tree, with the presence of PilT1 and PilT2 isoforms (Fig. S12) giving a maximum of eleven proteins evaluated in ten trees. However, missing or partial sequences reduced the actual number in most cases. ES-bin-313 and SpSt-379 had particularly poor overlap within this set (*n* = 6), so two possible topologies are indicated. Two topologies are also indicated for placement of early-branching, mesophilic members of the Thermostichales. For this meta-analysis, support at <70% was not considered robust (dashed lines). Y, node supported; N, node not supported; Ø, sequence not present.

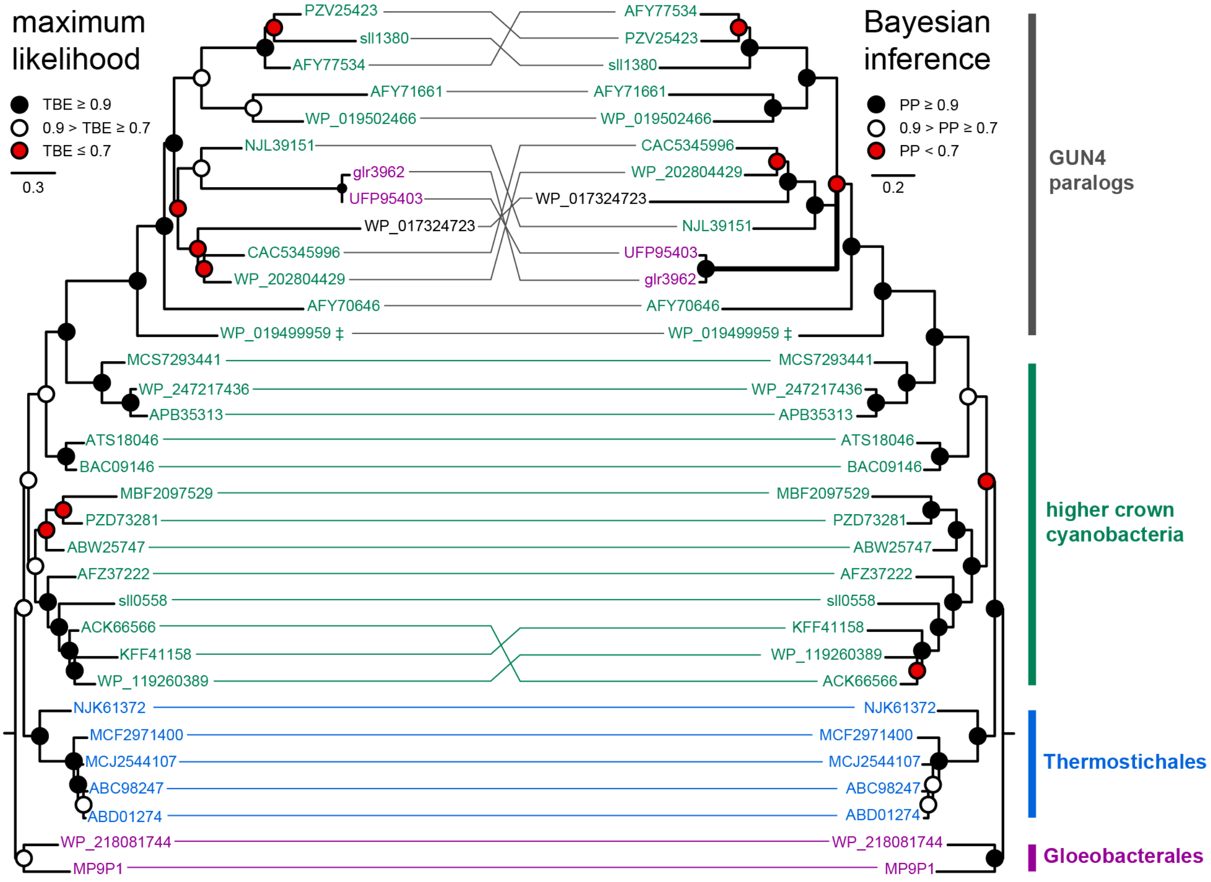

Fig. S15. Comparison of maximum likelihood and Bayesian phylogenetic analyses for cyanobacterial GUN4 proteins. A tanglegram representation is used to compare the maximum likelihood analysis of GUN4 (left; Fig. 5B) with a Bayesian analysis of the same alignment (right). ‡, incomplete sequence (defined as ≤90% of the expected length after gap removal). The thick line indicates a polytomy in the Bayesian topology. Taxa are indicated in Fig. 5.

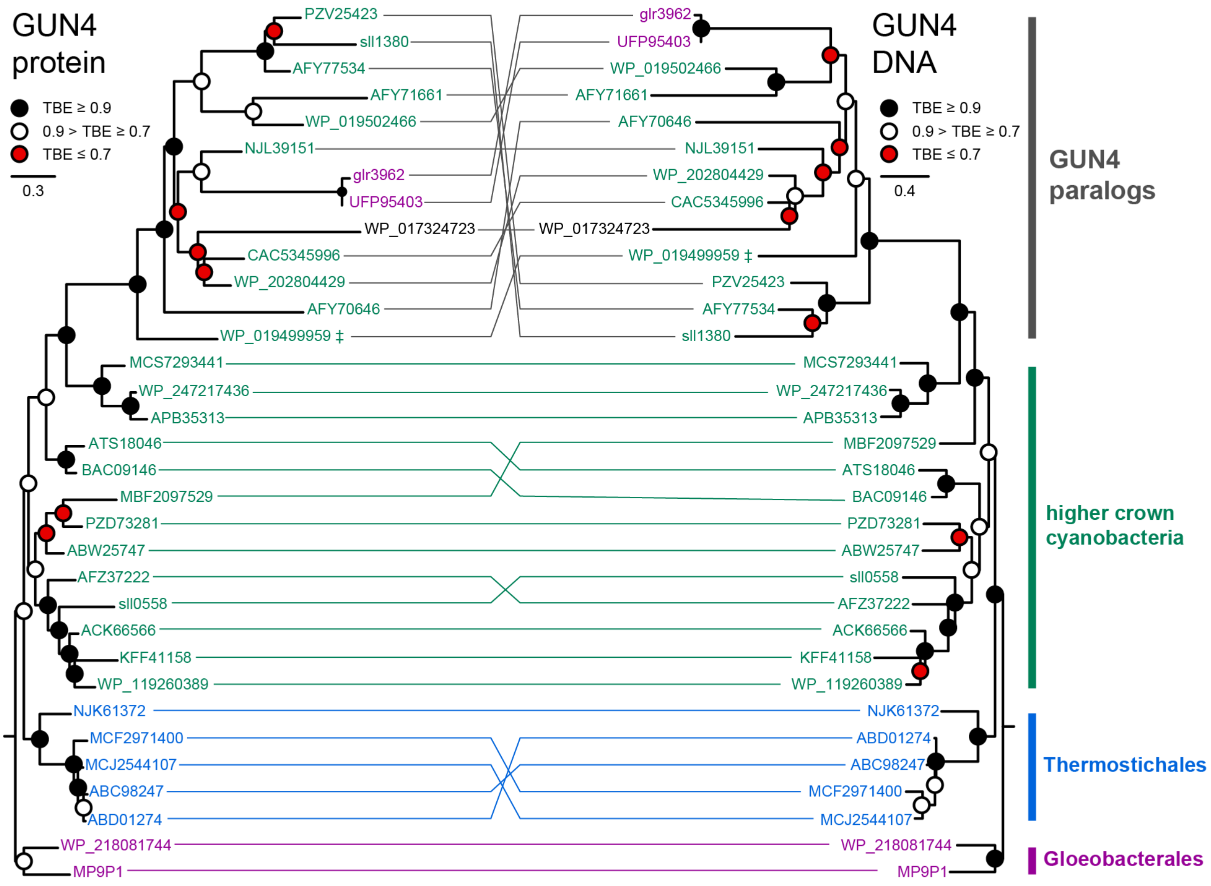

Fig. S16. Comparison of phylogenetic analysis of protein and DNA sequences for cyanobacterial GUN4 proteins. A tanglegram representation is used to compare the maximum likelihood analysis of GUN4 protein sequences (left; Fig. 5B) with a maximum likelihood analysis of the DNA sequences coding for the same proteins (right). ‡, incomplete sequence (defined as ≤90% of the expected length after gap removal). Taxa are indicated in Fig. 5.

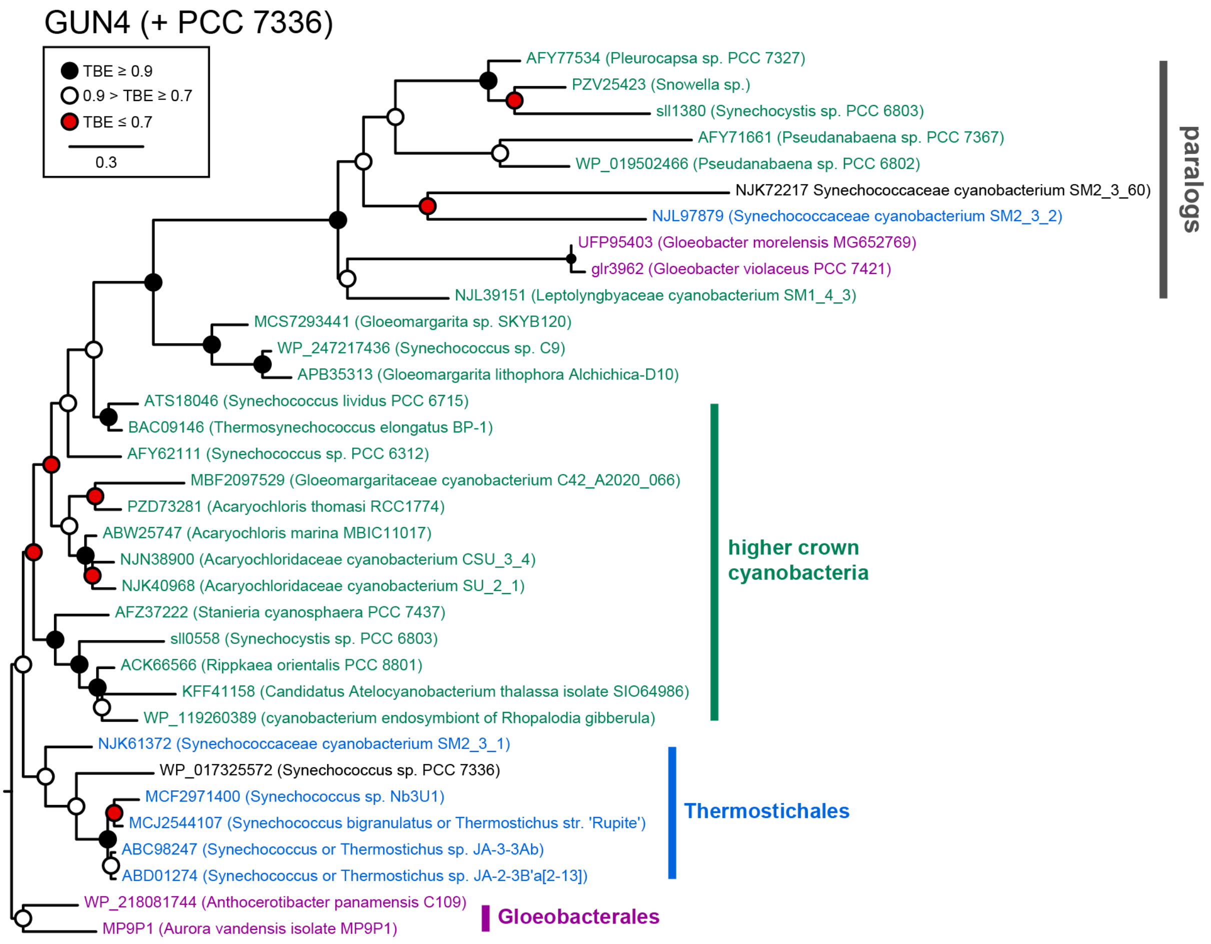

Fig. S17. Phylogenetic analysis of GUN4 Including the ortholog from *Synechococcus* sp. PCC 7336. A maximum-likelihood phylogenetic tree is shown for GUN4, using the conventions and root placement of Fig. 5B. This tree includes the GUN4 ortholog from *Synechocystis* PCC 7336 as well as additional paralogs. The G/T/HC topology is retained.

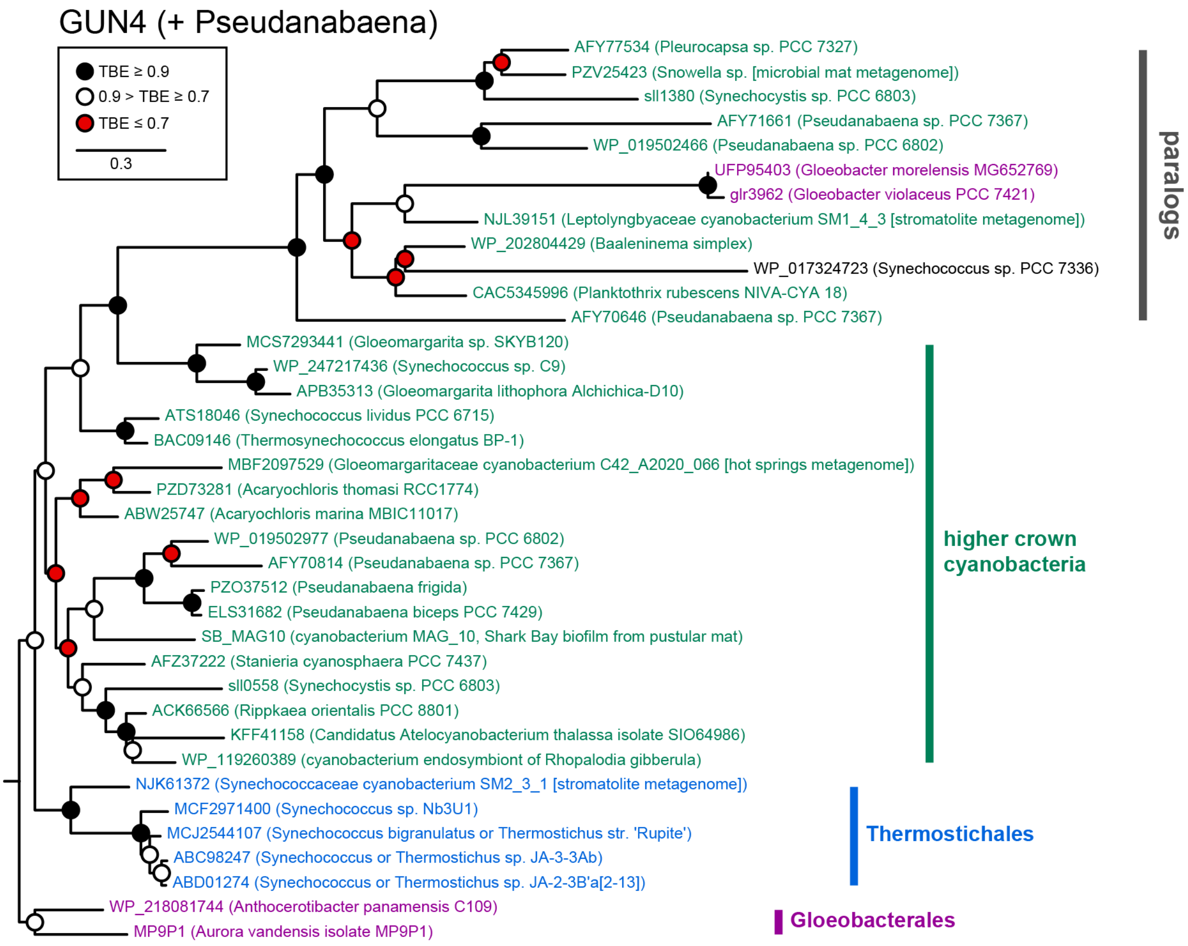

Fig. S18. Phylogenetic analysis of GUN4 including orthologs from *Pseudanabaena* spp. A maximum-likelihood phylogenetic tree is shown for GUN4, using the conventions and root placement of Fig. 5B. Relative to that figure, this tree includes GUN4 orthologs from *Pseudanabaena* spp. and an additional sequence reported from a microbial mat at Shark Bay, Australia (20). The G/T/HC topology is indicated.

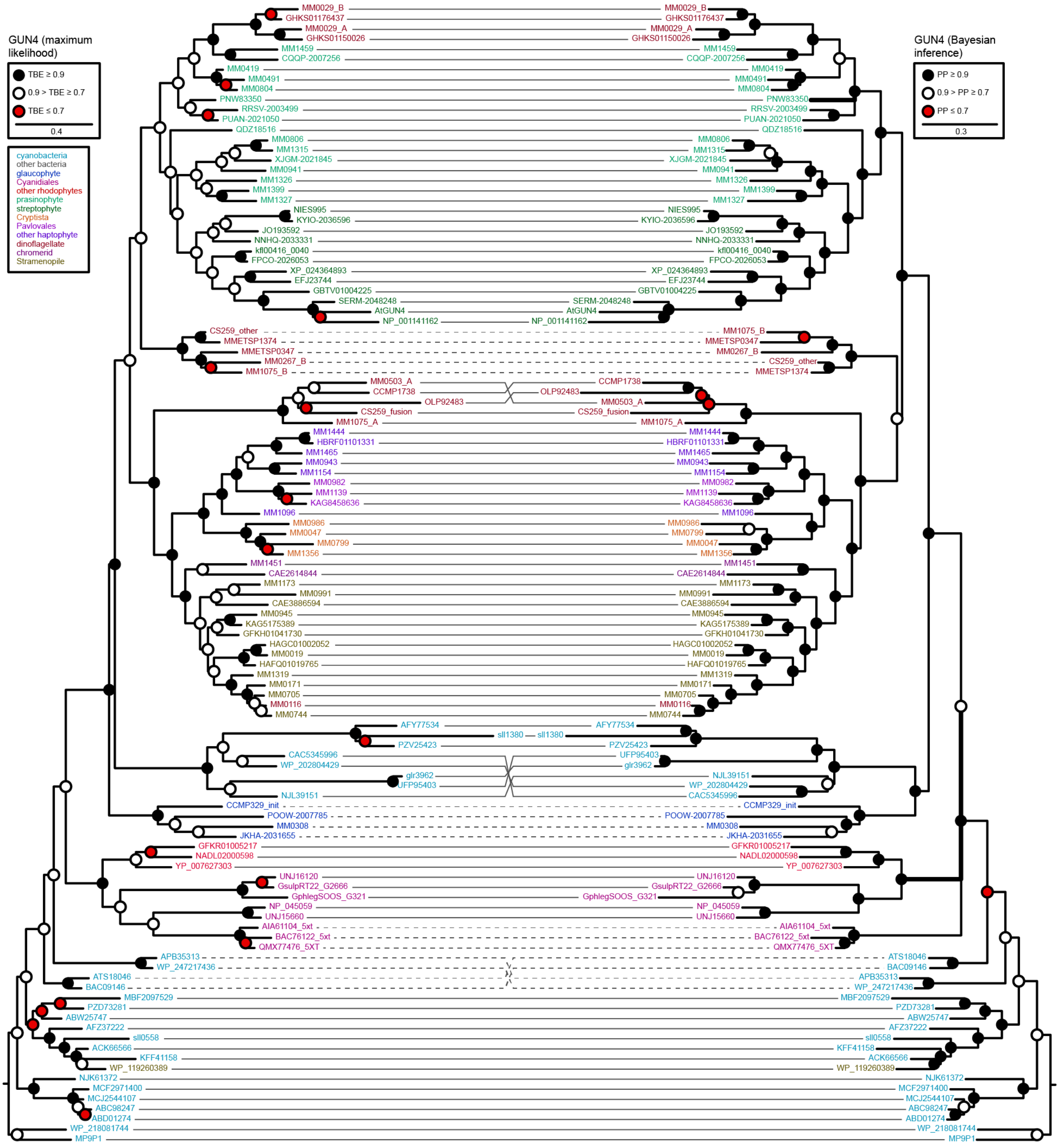

Fig. S19. Comparison of maximum likelihood and Bayesian phylogenetic analyses for GUN4 proteins. A tanglegram representation is used to compare the maximum likelihood analysis of cyanobacterial and eukaryotic GUN4 proteins (left; Fig. 9) with a Bayesian analysis of the same alignment (right). Thick lines indicate polytomies in the Bayesian topology, and dashed lines indicate clades that are placed differently in the two topologies. Taxa are listed in Fig. 9.

Table S1. Statistics for phylogenetic analyses

Target	Presented	Sequences	Characters	*n* ≤ 90% complete
16S rRNA	Fig. 2	88	1235	7
catenated ribosomal proteins	Fig. 3	45	1322	0
catenated ribosomal proteins	Fig. S3	44	684	0
ribosomal protein L2	Fig. S4	35	273	0
ribosomal protein S2	Fig. S5	37	237	0
Thr Synthase	Fig. S6	49	323	0
DPOR N subunit	Fig. S7	35	426	0
SufB	Fig. S8	31	477	0
subtilase	Fig. S9	44	313	0
PilM	Fig. S10	41	338	0
PilN	Fig. S11	37	177	0
PilT	Fig. S12	68	350	0
CP43	Fig. S13	32	438	0
GUN4	Figs. 5B, 6, S15, & S16	33	134	1
GUN4 (DNA)	Fig. S16	33	388	1
GUN4 (with PCC 7336)	Fig. S17	34	129	0
GUN4 (with *Pseudanabaena*)	Fig. S18	37	132	0
CHLH	Fig. 7	109	620	0
GUN4 (with eukaryotic sequences)	Fig. 9 & S19	113	143	0

Table S2. Ribosomal protein coverage in selected MAGs from Gloeobacterales and Thermostichales.

Protein	*T. elongatus*	SpSt-379	RM1_1_27	SM2_3_1	SM2_3_2	SM2_3_60
L3	BAC07634	HGZ88370			NJL99011	
L4	BAC07635	HGZ88371		NJK63764	NJL99012	
L2	BAC07637	HGZ88504			NJL99014	
S3	BAC07640				NJL99017	NJK72313
L16	BAC07641			NJK63768	NJL99018	NJK72312
L5	BAC07646			NJK63772	NJL99023	NJK72307
S8	BAC07647			NJK63773	NJL99024	NJK72306
L6	BAC07648			NJK63774		NJK72305
S5	BAC07650	HGZ84500		NJK63776	NJL99026	
L15	BAC07651	HGZ84501		NJK63777	NJL99027	
S11	BAC07657			NJK61430	NJL99042	
L13	BAC07661	HGZ88297		NJK61428	NJL99038	
S9	BAC07662	HGZ88296		NJK61427	NJL99037	
S4	BAC07700	HGZ86267		NJK64600	NJL99828	
L11	BAC07848		NJO85708	NJK63824	NJL97437	
L1	BAC07849	HGZ85908	NJO85707	NJK63825	NJL97438	NJK72725
S5	BAC07850	HGZ85907		NJK63826	NJL97439	
L15	BAC07851	HGZ85906	NJO85706	NJK61880	NJL97440	NJK72726
S2	BAC09240	HGZ85647	NJO85593	NJK61345	NJL99737	NJK72228
S12	BAC09299	HGZ87171		NJK62333	NJL97520	
S7	BAC09300	HGZ87172		NJK62334	NJL97519	fragment
S1	BAC09598	HGZ86502		NJK65104	NJL98897	NJK72501
L9	BAC09965	HGZ85111		NJK61472	NJL99367	

SI References

1. Y. Nakamura *et al.*, Complete genome structure of the thermophilic cyanobacterium Thermosynechococcus elongatus BP-1. *DNA Res.* **9**, 123-130 (2002).
2. S. F. Altschul *et al.*, Gapped BLAST and PSI-BLAST: a new generation of protein database search programs. *Nucleic Acids Res.* **25**, 3389-3402 (1997).
3. C. L. Grettenberger, Novel Gloeobacterales spp. from Diverse Environments across the Globe. *mSphere* **6**, e0006121 (2021).
4. J. H. Saw, T. Cardona, G. Montejano, Complete Genome Sequencing of a Novel Gloeobacter Species from a Waterfall Cave in Mexico. *Genome Biol. Evol.* **13** (2021).
5. K. Katoh, D. M. Standley, MAFFT: iterative refinement and additional methods. *Methods Mol. Biol.* **1079**, 131-146 (2014).
6. S. Guindon *et al.*, New algorithms and methods to estimate maximum-likelihood phylogenies: assessing the performance of PhyML 3.0. *Syst. Biol.* **59**, 307-321 (2010).
7. J. Mareš *et al.*, The Primitive Thylakoid-Less Cyanobacterium Gloeobacter Is a Common Rock-Dwelling Organism. *PLoS One* **8**, e66323 (2013).
8. P. M. Shih *et al.*, Improving the coverage of the cyanobacterial phylum using diversity-driven genome sequencing. *Proc. Natl. Acad. Sci. U.S.A.* **110**, 1053-1058 (2013).
9. R. I. Ponce-Toledo *et al.*, An Early-Branching Freshwater Cyanobacterium at the Origin of Plastids. *Curr. Biol.* **27**, 386-391 (2017).
10. Y. Hirose *et al.*, Diverse Chromatic Acclimation Processes Regulating Phycoerythrocyanin and Rod-Shaped Phycobilisome in Cyanobacteria. *Mol. Plant* **12**, 715-725 (2019).
11. K. R. Moore *et al.*, An Expanded Ribosomal Phylogeny of Cyanobacteria Supports a Deep Placement of Plastids. *Front. Microbiol.* **10**, 1612 (2019).
12. J. Komárek, J. R. Johansen, J. Šmarda, O. Strunecký, Phylogeny and taxonomy of Synechococcus–like cyanobacteria. *Fottea (Praha)* **20**, 171-191 (2020).
13. M. Y. Chen *et al.*, Comparative genomics reveals insights into cyanobacterial evolution and habitat adaptation. *ISME J.* **15**, 211-227 (2021).
14. G. P. Fournier *et al.*, The Archean origin of oxygenic photosynthesis and extant cyanobacterial lineages. *Proc. Royal Soc. B* **288**, 20210675 (2021).
15. N. Rahmatpour *et al.*, A novel thylakoid-less isolate fills a billion-year gap in the evolution of Cyanobacteria. *Curr. Biol.* **31**, 2857-2867 e2854 (2021).
16. O. Strunecký, A. P. Ivanova, J. Mareš, An updated classification of cyanobacterial orders and families based on phylogenomic and polyphasic analysis. *J. Phycol.* **59**, 12-51 (2023).
17. D. Konstantinou, E. Voultsiadou, E. Panteris, S. Gkelis, Revealing new sponge-associated cyanobacterial diversity: Novel genera and species. *Mol. Phylogenet. Evol.* **155**, 106991 (2021).
18. N. C. Rockwell, D. J. Krysan, T. Komiyama, R. S. Fuller, Precursor processing by kex2/furin proteases. *Chem. Rev.* **102**, 4525-4548 (2002).
19. B. Khayatan, J. C. Meeks, D. D. Risser, Evidence that a modified type IV pilus-like system powers gliding motility and polysaccharide secretion in filamentous cyanobacteria. *Mol. Microbiol.* **98**, 1021-1036 (2015).
20. E. J. Skoog *et al.*, Metagenomic, (bio)chemical, and microscopic analyses reveal the potential for the cycling of sulfated EPS in Shark Bay pustular mats. *ISME commun.* **2**, 43 (2022).
